# Supplementary material for: Loneliness is associated with unstable and distorted emotion transition predictions
Source: Commun Psychol. 2025 Aug 28;3:132. doi: 10.1038/s44271-025-00310-w (PMC12394594; doi:10.1038/s44271-025-00310-w)
Supplement: Supplementary file 2 — Supplemental Material [file 44271_2025_310_MOESM2_ESM.pdf]

Supplementary Information for **"Loneliness is associated with unstable and distorted emotion transition predictions"**

Ava Q. Ma de Sousa[1]*, Miriam E. Schwyck[2], Laura Furtado Fernandes[3], Ezra Ford[3], Begüm G. Babür[5], Chang Lu[5], Jacob C. Zimmerman[5], Hongbo Yu[1], Shannon M. Burns[3,4]*, Elisa C. Baek[5]*

[1]Department of Psychological and Brain Sciences, University of California, Santa Barbara, Santa Barbara CA, United States of America,

[2]Department of Psychology, Columbia University, New York, NY, United States of America,

[3]Department of Psychological Science, Pomona College, Pomona, CA, United States of America,

[4]Department of Neuroscience, Pomona College, Pomona, CA, United States of America,

[5]Department of Psychology, University of Southern California, Los Angeles, CA, United States of America

*Corresponding authors

Supplementary Table 1

Emotion categorizations across studies

Study	Positive Emotions	Neutral	Negative emotions
Study 1	calm, happy	full of thought	anxious, irritable, sad, sluggish
Study 2	calm, happy	full of thought	anxious, irritable, sad, sluggish
Study 3	calm, happy	full of thought	anxious, irritable, sad, sluggish
Study 4	confident, satisfaction, love, lively, bold, unrestrained, talkative, assertive	N/A	sad, irritable, nervous, contempt, disgust, embarrassment, grouchy
Study 5	calm, happy	alert	anxious, irritable, sad, sluggish
Study 6	content, amused, hopeful, satisfied	N/A	anxious, sad
Study 7	content, amused, hopeful, satisfied	N/A	anxious, sad

Supplementary Table 2

Shapiro-Wilk and Breusch-Pagan Tests for Typicality Models

	Self		Other	
**Study**	**Shapiro-Wilk W**	**Breusch-Pagan χ^2**	**Shapiro-Wilk W**	**Breusch-Pagan χ^2**
Study 1	0.782***	1.501	0.780***	1.35
Study 2	0.861***	6.672**	0.843***	6.651**
Study 3	0.804***	4.707*	0.893***	11.919***
Study 4	0.928***	10.097**	0.875***	9.459**
Study 5	0.898***	0.001	0.882***	0.573
Study 6	0.909**	0.008	0.947	0.094
Study 7	0.945***	0.223	—	—

Note. * $p < .05$. ** $p < .01$. *** $p < .001$.

Supplementary Table 3
DHARMa Diagnostics for Accuracy Models

Study	KS D	Dispersion Ratio
Study 1	0.042***	0.999
Study 2	0.037***	0.999
Study 3	0.024***	1.004
Study 4	0.043***	1.000
Study 5	0.022	0.999
Study 6	0.028	0.990

Note. * $p < .05$. ** $p < .01$. *** $p < .001$.

Supplementary Table 4
DHARMa Diagnostics for Anchoring Models

Study	KS D	Dispersion Ratio
Study 1	0.027***	1.001
Study 2	0.019**	0.999
Study 3	0.042***	0.999
Study 4	0.031***	1.00
Study 5	0.031**	0.980
Study 6	0.045*	0.982

Note. * $p < .05$. ** $p < .01$. *** $p < .001$.

Supplementary Table 5
DHARMa Diagnostics for Valence Models

	Self	Other		Self
Study	KS D	Dispersion Ratio	KS D	Dispersion Ratio
Study 1	0.040***	1.00	0.028**	0.995
Study 2	0.033***	1.00	0.025***	0.995
Study 3	0.026***	0.994	0.028***	0.995
Study 4	0.029***	0.999	0.027***	0.997
Study 5	0.043***	0.990	0.027*	0.990
Study 6	0.052**	0.985	0.057***	0.987

Note. * $p < .05$. ** $p < .01$. *** $p < .001$.

Supplementary Table 6

Shapiro-Wilk and Breusch-Pagan Tests for Confidence Models

	Self		Other	
Study	Shapiro-Wilk W	Breusch-Pagan χ^2	Shapiro-Wilk W	Breusch-Pagan χ^2
Study 1	0.977*	1.636	0.984	0.402
Study 2	0.976**	0.869	0.978**	6.988**
Study 3	0.998	0.004	0.998	2.221
Study 4	0.977	7.930**	0.989	7.866**
Study 5	0.988	1.937	0.987	1.937
Study 6	0.969	0.078	0.966	0.445
Study 7	0.987***	2.436	—	—

*Note. * p < .05. ** p < .01. *** p < .001.*

Typicality Results by Study for Self and Other

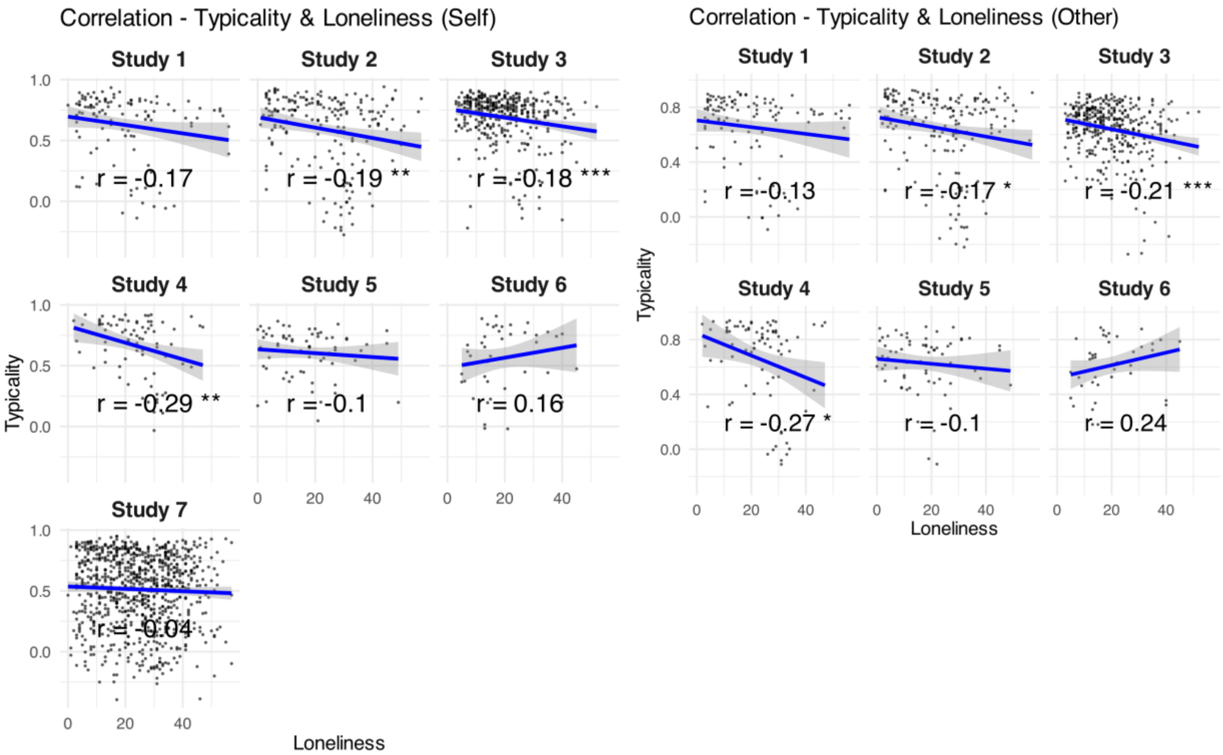

Supplementary Fig. 1

Correlations of typicality and Loneliness for Self (left) and Other (right)

Pearson correlations for the relationship between typicality and Loneliness for self and other ratings. *p* values are indicated as follows: *** $p < .001$, **$p < .01$, *$p < .05$.

Typicality Results Controlling for Age

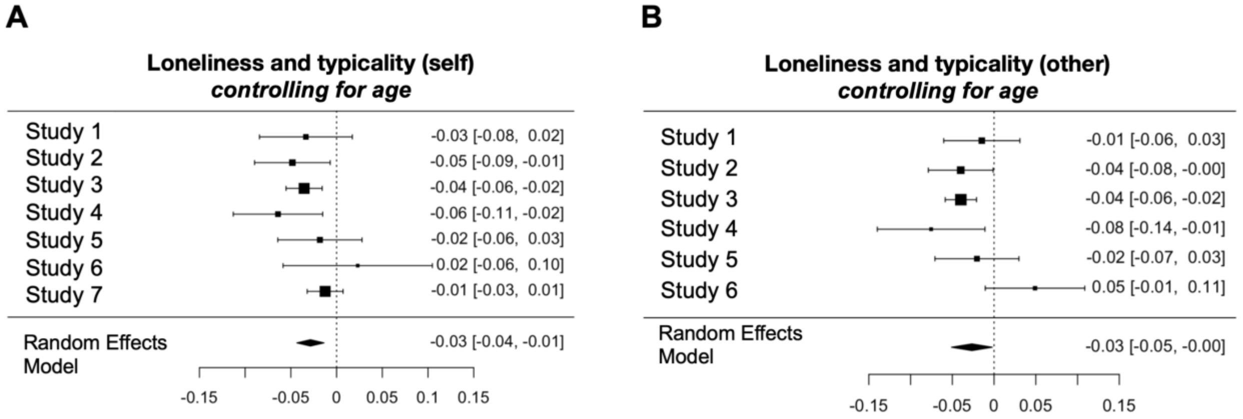

Supplementary Fig. 2

Meta-analytic Effects of the Interaction Between Typicality and Loneliness, controlling for age

(A) Partial correlation estimates relating typicality and loneliness for self ratings, controlling for age, for each study are visualized. (**B**) Partial correlation estimates relating typicality and loneliness for other ratings, controlling for age, are visualized. Each square represents the correlation estimate from a single dataset with error bars denoting the corresponding 95% confidence intervals. The rhombus at the bottom represents the overall meta-analytic correlation estimate with its midpoint indicating the average effect size and its width representing the 95% confidence interval, summarizing the effect size across all included studies.

Transition-Level Typicality

We conducted additional analyses to explore which particular emotion transitions may be driving our observed effects relating loneliness and atypical predictions. To do so, we focused on data from Studies 1-3, as they were the only studies that used the same set of emotions. For each transition, we calculated a typicality score by taking the absolute difference between an individual's rating and the corresponding group mean. Larger values therefore represent ratings that diverge further from the normative pattern. For each transition, we then correlated loneliness with this typicality score (two-tailed, $p < .05$, uncorrected).

Self transition ratings. Loneliness was associated with larger deviations from the group norm on several transitions that involved maintaining or moving away from positive states (Supplementary Fig. 3A). In particular, greater loneliness predicted larger deviations for the

transitions calm to calm ($r = .087$, $p = .025$), calm to happy ($r = .112$, $p = .004$), full of thought to happy ($r = .113$, $p = .003$), happy to happy ($r = .158$, $p < .001$), happy to irritated ($r = .101$, $p = .009$), and happy to sad ($r = .129$, $p < .001$).

Other transition ratings. We repeated the same procedure for participants' estimates of how *others* transition between emotions. Again, higher loneliness was tied to less typical judgments for several ratings (Supplementary Fig. 3B): anxious to happy ($r = .086$, $p = .025$), calm to irritated ($r = .091$, $p = .019$), happy to happy ($r = .088$, $p = .023$), sad to anxious ($r = .079$, $p = .041$), sad to irritated ($r = .086$, $p = .026$), sad to sluggish ($r = .093$, $p = .015$), and sluggish to happy ($r = .092$, $p = .017$).

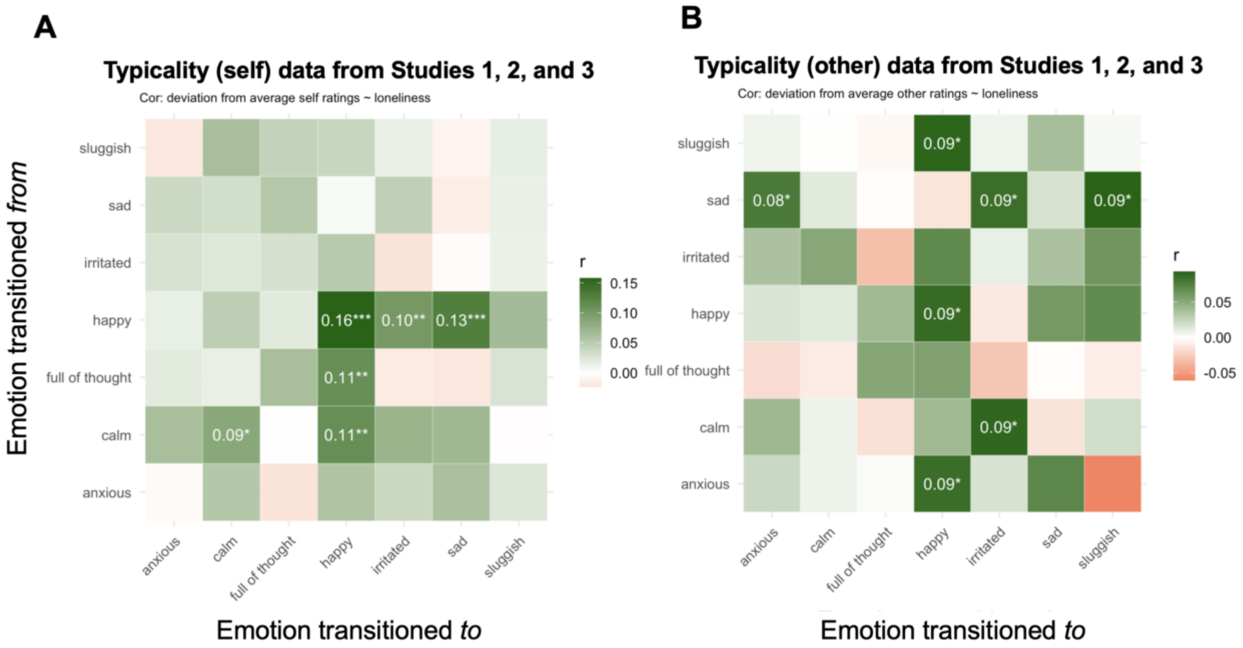

Supplementary Fig. 3
(A) *Heatmap relating typicality and loneliness by specific emotion transition type from combined data of Study 1, 2, and 3.* Positive correlations indicate that loneliness is associated with stronger deviations in ratings from the group average (i.e. atypicality). Uncorrected p values are indicated as follows: *** $p < .001$, **$p < .01$, *$p < .05$.
(B) *Heatmap relating typicality and loneliness by specific emotion transition type from combined data of Study 1, 2, and 3.* Positive correlations indicate that loneliness is associated with stronger deviations in ratings from the group average (i.e., atypicality). Uncorrected p values are indicated as follows: *** $p < .001$, **$p < .01$, *$p < .05$.

"Anna Karenina" Typicality Analysis

To further probe the associations between loneliness and atypicality of emotion transition, we took an "Anna Karenina" approach[1,2]. Specifically, we tested whether non-lonely individuals are all alike, but every lonely individual holds expectations of emotion transitions in their own idiosyncratic way.

To do this, we conducted inter-subject representational-similarity analysis (IS-RSA) to participants' estimates of emotion-transition likelihoods. For each study, we calculated the Pearson correlation between every pair of participants' rating vectors to obtain a participant-by-participant similarity matrix. We did this twice, once for self ratings and once for other ratings. Next, we constructed a predictor matrix that embodied the "Anna Karenina" ("AnnaK") principle for loneliness. For each pair of participants, we averaged their two ranks based on their loneliness score, with higher averaged ranks representing pairs in which at least one member is highly lonely. Following the approach of Finn et al. (2020), representational similarity was assessed by calculating Spearman's correlation of the vectorized upper-triangular portions of the emotion transition ratings similarity matrix and the loneliness AnnaK matrices (excluding diagonals). A negative ρ means that the more loneliness a pair contains, the less alike their emotion-transition ratings are.

The resulting ρ coefficient captures the degree to which the pattern of (dis)similarity dictated by loneliness explains the pattern of (dis)similarity in emotion-transition space: thus, a negative value indicates that the more lonely the members of a dyad are, the more dissimilar they are. Finally, as in other analyses, we then conducted an internal meta-analyses to obtain an overall estimate of the association between loneliness and typicality of ratings for self and other, respectively.

Self ratings. Results indicate support for the Anna Karenina pattern: non-lonely individuals were similar to one another in their self transition ratings, while lonely individuals were idiosyncratic ($\rho = -0.135$, SE = 0.044, $p = .002$, 95% CI [-0.221, -0.048], $\tau^2 = 0.007$, $I^2 = 57.83\%$). In other words, lonely individuals were atypical in their ratings for self transitions in their own unique way, in ways that are different from not only non-lonely individuals but also from other lonely individuals.

Other ratings. Results again support the Anna Karenina pattern: non-lonely individuals were similar to one another in their transition ratings for others, whereas lonely individuals were idiosyncratic ($\rho = -0.163$, SE = 0.033, $p < 0.001$, 95% CI [-0.228, -0.097], $\tau^2 < 0.001$, $I^2 = 0.00\%$). Here again, lonely individuals were atypical in their ratings for others' transitions, in ways that were different from both non-lonely individuals and other lonely individuals.

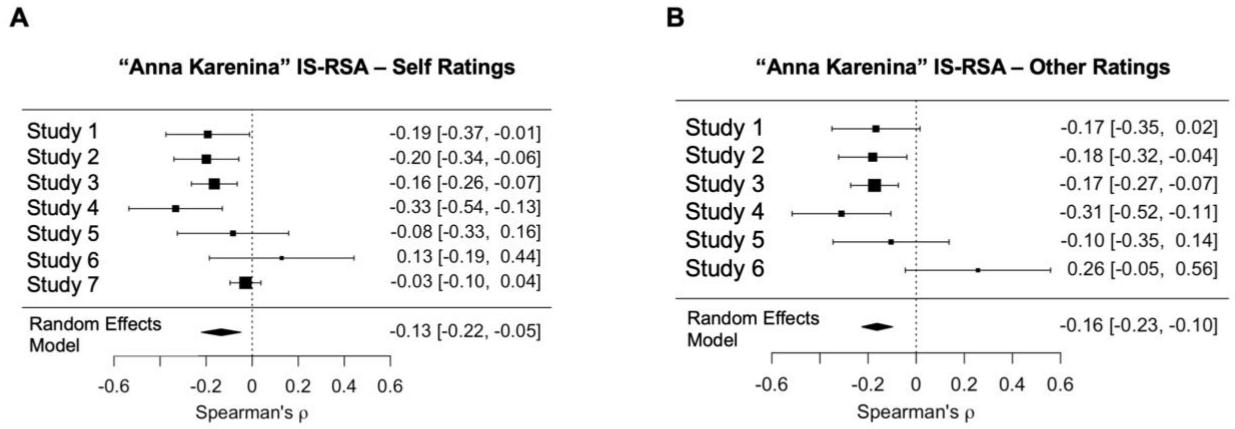

Supplementary Fig. 4
"Anna Karenina" intersubject RSA
Individual study estimates of the "Anna Karenina" models for (A) self transition ratings and (B) other transition ratings are visualized. Across both panels, each square represents the estimated intersubject correlation (Spearman's rho) from a single dataset, with error bars indicating the corresponding 95% confidence intervals. The rhombus at the bottom represents the overall meta-analytic estimate, with its midpoint indicating the pooled effect size and its width representing the 95% confidence interval, summarizing the effect across all included studies. A negative value corresponds to lonely individuals displaying more idiosyncratic ratings (i.e., patterns different from non-lonely people and other lonely people), and less lonely people converging in their ratings.

Accuracy Results: Main Effects by Study

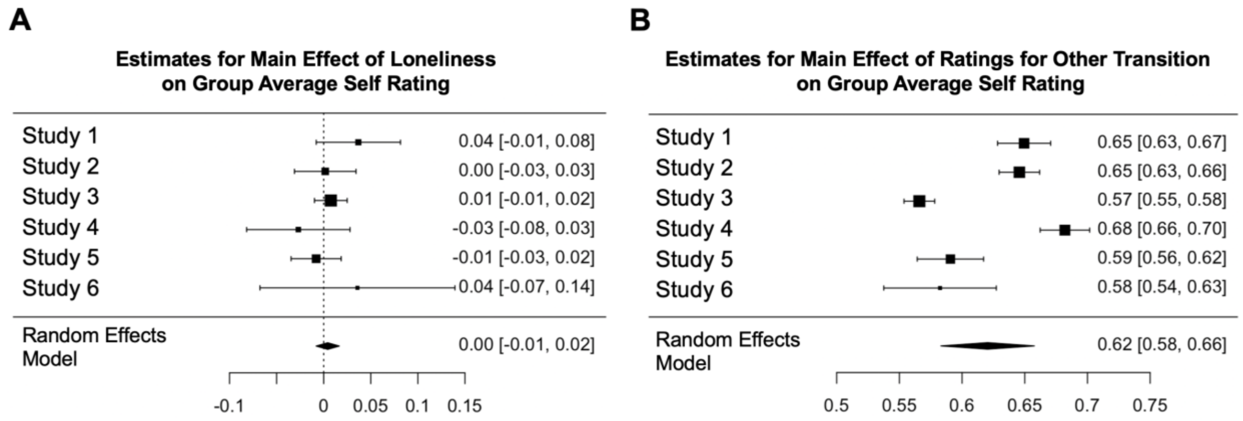

Supplementary Fig. 5

Meta-analytic Main effects of Accuracy Analysis

Individual study estimates for (A) the main effects of loneliness and (B) the main effects of other transition ratings are visualized. Across both panels, each square represents the estimated effect from a single dataset, with error bars indicating the corresponding 95% confidence intervals. The rhombus at the bottom represents the overall meta-analytic estimate, with its midpoint indicating the pooled effect size and its width representing the 95% confidence interval, summarizing the effect across all included studies. (A) Effect sizes close to zero indicate that participant loneliness does not predict group average self ratings, as expected. (B) Positive effect sizes show that participants' ratings of others' emotion transitions predict group average self transition ratings.

Accuracy Results: Interaction Effects by Study

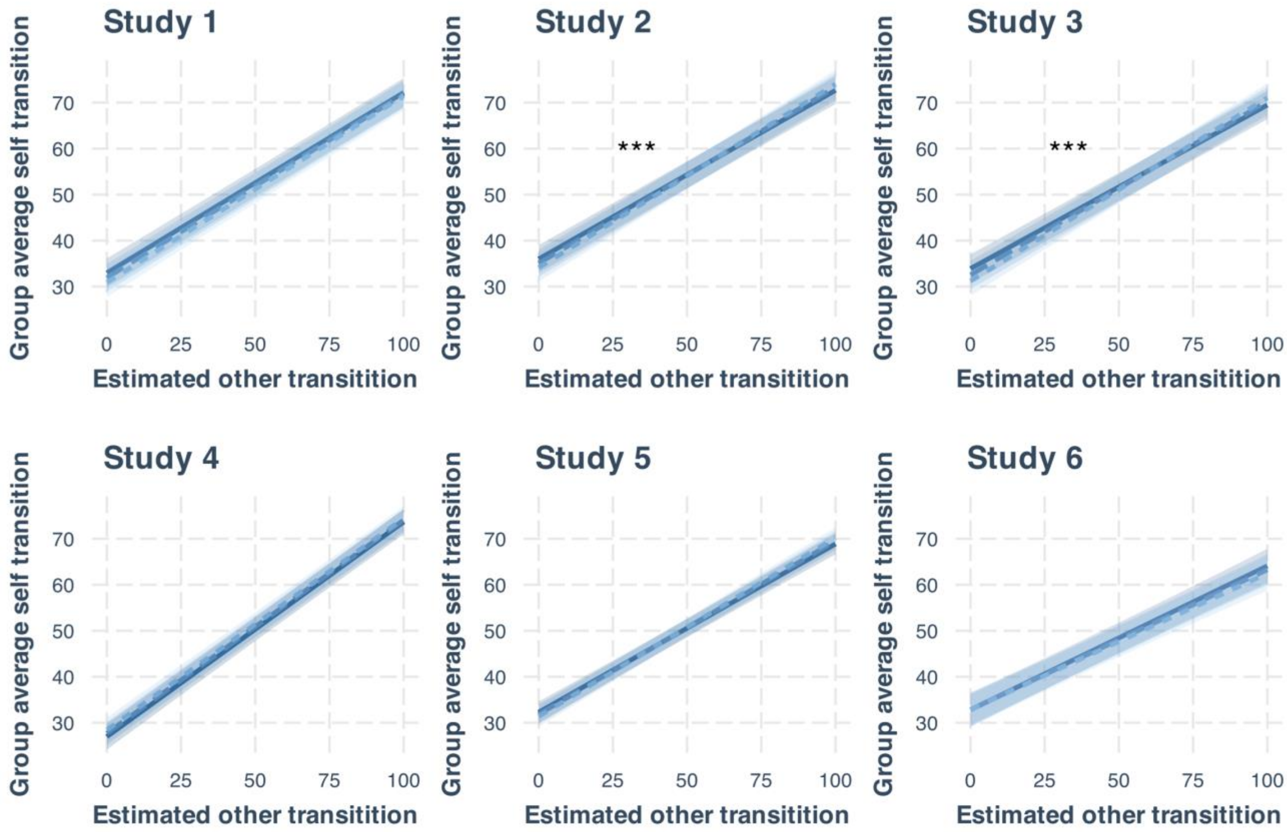

Supplementary Fig. 6

Accuracy Results By Study

Linear mixed-effects models for all datasets. p values for interactions between estimated likelihood of other's emotion transition and loneliness are indicated as follows: *** $p < .001$, **$p < .01$, *$p < .05$.

Supplementary Table 7

Accuracy of Estimated Transition Likelihood for Others Across Studies

	Study 1	*Study 2*	*Study 3*	*Study 4*	*Study 5*	*Study 6*
Predictors	*Estimate (95% CI)*	*Estimate (95% CI)*	*Estimate (95% CI)*	*Estimate (95% CI)*	*Estimate (95% CI)*	*Estimate (95% CI)*
(Intercept)	53.305 *** (50.399 – 56.212)	56.604 *** (53.731 – 59.476)	54.011 *** (50.975 – 57.047)	54.062 *** (51.709 – 56.414)	51.871 *** (49.959 – 53.783)	46.238 *** (43.034 – 49.442)
Estimated Other-transition (c)	0.399 *** (0.386 – 0.412)	0.383 *** (0.373 – 0.392)	0.377 *** (0.369 – 0.385)	0.463 *** (0.450 – 0.477)	0.377 *** (0.360 – 0.394)	0.332 *** (0.306 – 0.358)
Loneliness (c)	0.049 (-0.011 – 0.109)	0.002 (-0.040 – 0.044)	0.014 (-0.018 – 0.045)	-0.051 (-0.156 – 0.053)	-0.013 (-0.062 – 0.037)	0.039 (-0.072 – 0.150)
Estimated Other-transition (c) * Loneliness (c)	-0.000 (-0.001 – 0.000)	-0.001 *** (-0.002 – -0.001)	-0.002 *** (-0.003 – -0.001)	0.000 (-0.001 – 0.002)	-0.001 (-0.002 – 0.000)	0.002 (-0.000 – 0.005)
**Random Effects**						
σ^2	188.24	160.85	187.51	184.76	167.40	129.04
τ_{00}	15.94 Participant	12.14 Participant	5.37 Participant	20.59 Participant	2.18 Participant	9.94 Participant
	5.56 fromEmotion	5.68 fromEmotion	12.13 fromEmotion	13.18 fromEmotion	2.80 fromEmotion	5.65 fromEmotion
	8.60 toEmotion	8.77 toEmotion	4.49 toEmotion	4.15 toEmotion	3.28 toEmotion	8.26 toEmotion
ICC	0.14	0.14	0.10	0.17	0.05	0.16
N	113 Participant	185 Participant	376 Participant	81 Participant	68 Participant	41 Participant
	7 fromEmotion	7 fromEmotion	7 fromEmotion	15 fromEmotion	7 fromEmotion	6 fromEmotion
	7 toEmotion	7 toEmotion	7 toEmotion	15 toEmotion	7 toEmotion	6 toEmotion
Observations	5537	9065	18421	6075	3479	1204
Marginal R^2 / Conditional R^2	0.396 / 0.479	0.403 / 0.487	0.325 / 0.396	0.433 / 0.530	0.366 / 0.396	0.340 / 0.443

* *p<0.05* ** *p<0.01* *** *p<0.001*

Linear mixed-effects models predicting estimated transition likelihood for others based on estimated transition likelihood (centered), loneliness (centered), and their interaction. Random effects include variance components at the participant, from-emotion, and to-emotion levels. Intraclass correlations (ICCs) indicate the proportion of variance attributable to these levels. The number of participants, from-emotion, and to-emotion levels, as well as total observations, are provided for each study. Marginal and conditional R^2 values represent the variance explained by fixed effects alone and by the full model, respectively. Significance levels: *$p < .05$, **$p < .01$, ***$p < .001$.

Accuracy Results Controlling for Age

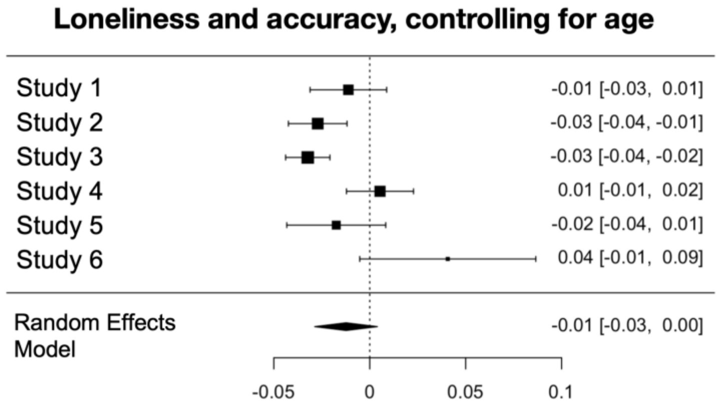

Supplementary Fig. 7
Meta-analytic Effects of Accuracy and Loneliness, controlling for age
Effect size estimates of the relationship between loneliness and accuracy for other ratings for each study controlling for age are visualized. Each square represents the correlation estimate from a single dataset with error bars denoting the corresponding 95% confidence intervals. The rhombus at the bottom represents the overall meta-analytic correlation estimate with its midpoint indicating the average effect size and its width representing the 95% confidence interval, summarizing the effect size across all included studies.

Transition-Level Accuracy

We conducted additional analyses to explore which particular emotion transitions may be driving our observed effects relating loneliness and accuracy. To do so, we focused on data from Studies 1–3, as they were the only studies that used the same set of emotions.

Here, we operationalized accuracy for each transition as the absolute difference between a participant's ratings for others and our "ground truth" proxy (i.e., group average transition ratings for self) and correlated this absolute difference measure with loneliness for each transition type. Positive correlations indicate that higher loneliness is associated with poorer accuracy (larger error) for that transition. We found that loneliness was associated with *less* accuracy in transition estimates in transitions (at p <.05, uncorrected) from 1) anxious to happy (r = 0.091, p = .018), 2) calm to irritated (r = 0.100, p = .009), 3) happy to happy (r = 0.108, p = .005), 4) happy to sluggish (r = 0.091, p = .018), 5) irritated to happy (r = 0.077, p = .045), 6)

sad to irritated ($r = 0.086$, $p = .026$), 7) sad to sluggish ($r = 0.086$, $p = .026$), 8) sluggish to happy ($r = 0.095$, $p = .014$) (See Supplementary Fig. 8).

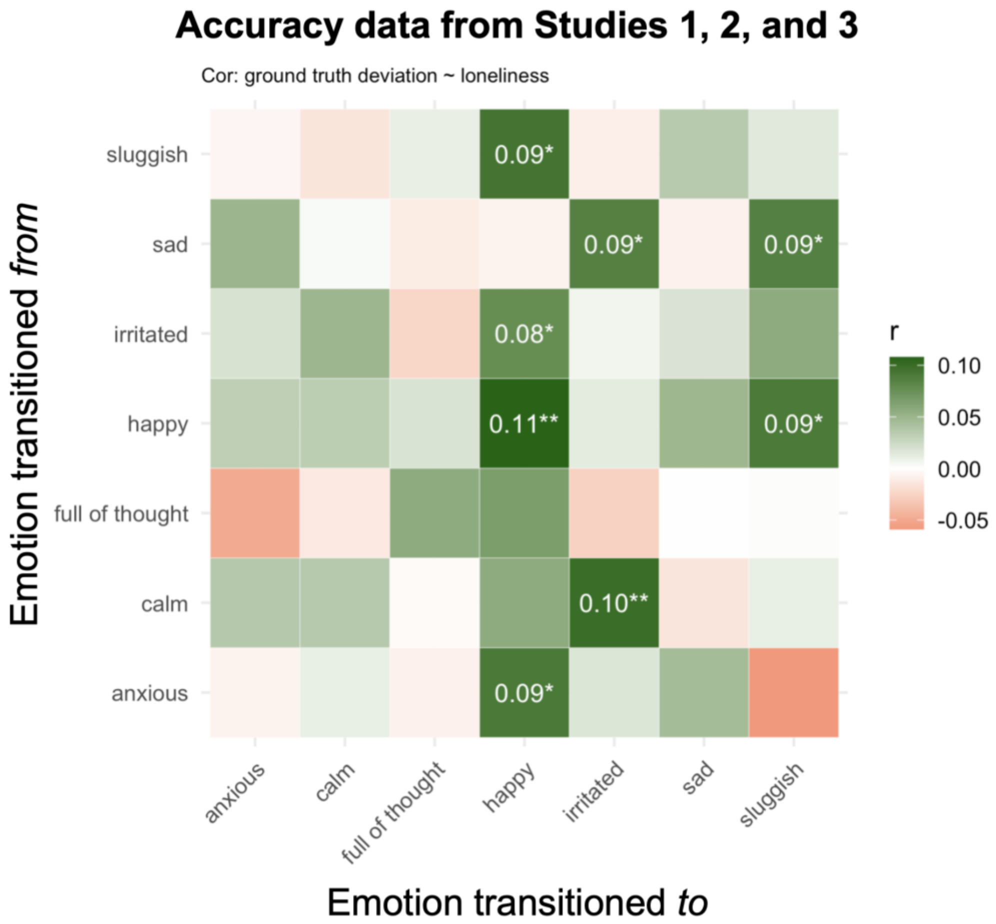

Supplementary Fig. 8
Heatmap of accuracy and loneliness by specific emotion transition type, from combined data of Study 1, 2, and 3. Positive correlations indicate that loneliness is associated with stronger deviations in ratings from the group average self rating (i.e., reduced accuracy). Uncorrected p values are indicated as follows: *** $p < .001$, **$p < .01$, *$p < .05$.

Anchoring Results: Main Effects by Study

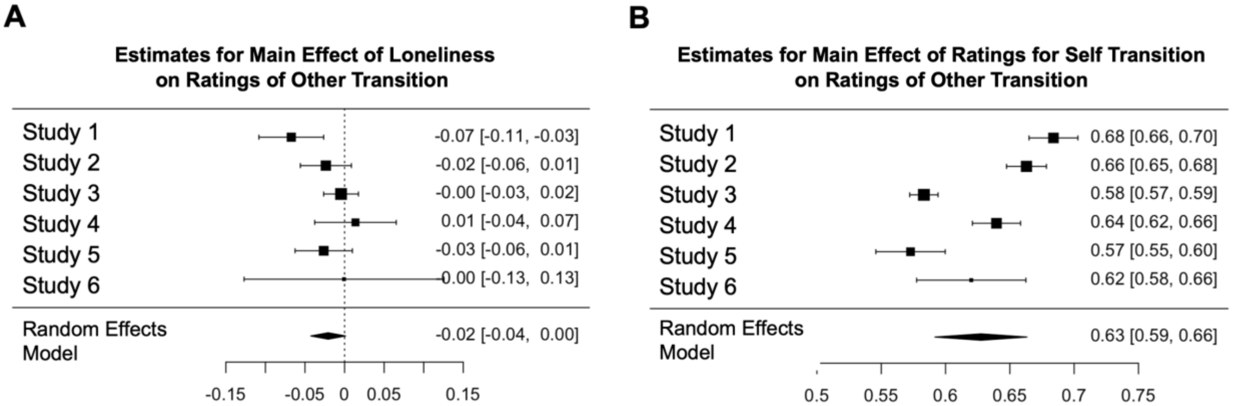

Supplementary Fig. 9

Meta-analytic main effects of loneliness and anchoring on the self to predict others

Individual study estimates for (A) the main effects of loneliness and (B) the main effects of self transition ratings are visualized. Across both panels, each square represents the estimated effect from a single dataset, with error bars indicating the corresponding 95% confidence intervals. The rhombus at the bottom represents the overall meta-analytic estimate, with its midpoint indicating the pooled effect size and its width representing the 95% confidence interval, summarizing the effect across all included studies. (A) Negative effect sizes indicate that loneliness is related to overall lower transition likelihood expectations for others. (B) Positive effect sizes indicate that participants' ratings of their own (self) emotion transitions predict their ratings of others' emotion transitions.

Anchoring Results: Interaction Effects by Study

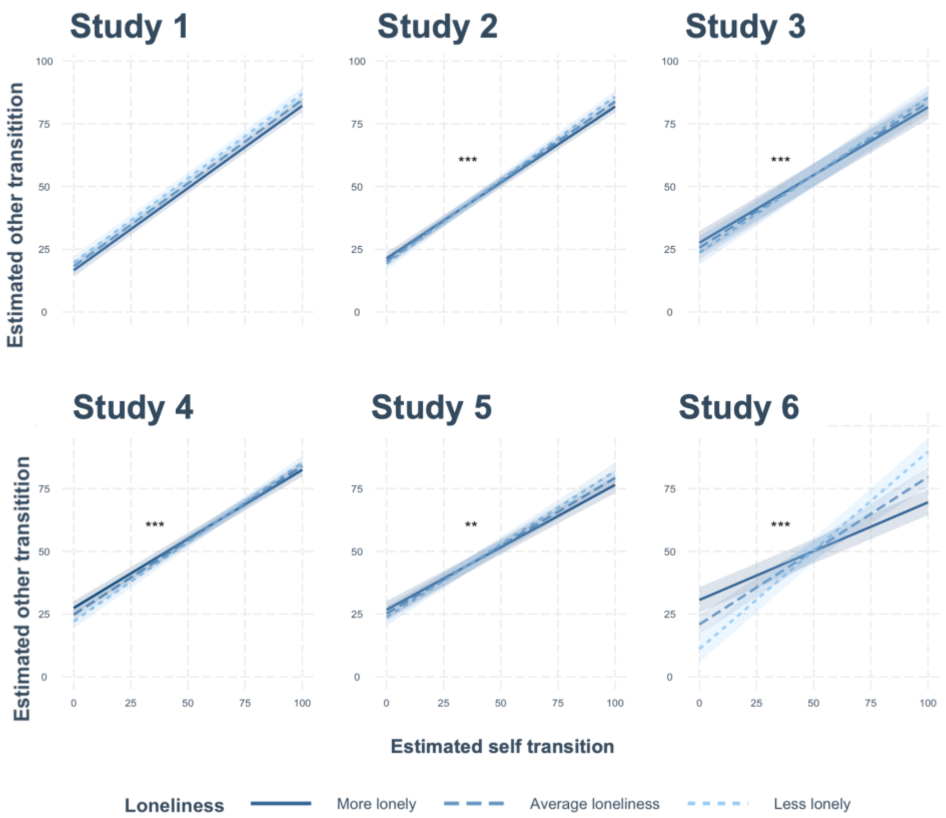

Supplementary Fig. 10

Anchoring Results by Study

Linear mixed-effects models for all datasets. *p* values for interactions between estimated likelihoods of self transition and loneliness are indicated as follows: *** $p < .001$, **$p < .01$, *$p < .05$.

Supplementary Table 8

Anchoring Effects in Estimated Transition Likelihood for Others Across Studies

	Study 1	*Study 2*	*Study 3*	*Study 4*	*Study 5*	*Study 6*
Predictors	*Estimate (95% CI)*	*Estimate (95% CI)*	*Estimate (95% CI)*	*Estimate (95% CI)*	*Estimate (95% CI)*	*Estimate (95% CI)*
(Intercept)	53.572 *** (51.759 – 55.385)	56.288 *** (54.443 – 58.132)	56.877 *** (52.255 – 61.499)	56.939 *** (54.744 – 59.133)	53.236 *** (50.337 – 56.135)	48.364 *** (45.894 – 50.835)
Self-transition (c)	0.664 *** (0.646 – 0.682)	0.636 *** (0.621 – 0.651)	0.579 *** (0.568 – 0.590)	0.598 *** (0.580 – 0.615)	0.544 *** (0.519 – 0.570)	0.594 *** (0.552 – 0.636)
Loneliness (c)	-0.147 ** (-0.236 – -0.058)	-0.051 (-0.121 – 0.019)	-0.012 (-0.071 – 0.047)	0.039 (-0.106 – 0.183)	-0.061 (-0.146 – 0.023)	-0.027 (-0.261 – 0.208)
Self-transition (c) * Loneliness (c)	-0.001 (-0.002 – 0.001)	-0.002 *** (-0.003 – -0.001)	-0.004 *** (-0.005 – -0.003)	-0.005 *** (-0.006 – -0.003)	-0.004 ** (-0.006 – -0.001)	0.002 (-0.002 – 0.007)
**Random Effects**						
σ^2	409.52	420.54	362.81	380.87	447.15	369.60
τ_{00}	35.55 $_{Participant}$	34.14 $_{Participant}$	25.70 $_{Participant}$	38.92 $_{Participant}$	7.18 $_{Participant}$	50.41 $_{Participant}$
	2.98 $_{fromEmotion}$	3.92 $_{fromEmotion}$	1.06 $_{fromEmotion}$	0.98 $_{fromEmotion}$	4.92 $_{fromEmotion}$	0.18 $_{fromEmotion}$
	0.29 $_{toEmotion}$	0.67 $_{toEmotion}$	37.25 $_{toEmotion}$	9.67 $_{toEmotion}$	8.72 $_{toEmotion}$	0.04 $_{toEmotion}$
ICC	0.09	0.08	0.15	0.12	0.04	0.12
N	113 $_{Participant}$	185 $_{Participant}$	376 $_{Participant}$	81 $_{Participant}$	68 $_{Participant}$	41 $_{Participant}$
	7 $_{fromEmotion}$	7 $_{fromEmotion}$	7 $_{fromEmotion}$	15 $_{fromEmotion}$	7 $_{fromEmotion}$	6 $_{fromEmotion}$
	7 $_{toEmotion}$	7 $_{toEmotion}$	7 $_{toEmotion}$	15 $_{toEmotion}$	7 $_{toEmotion}$	6 $_{toEmotion}$
Observations	5537	9065	18419	6075	3479	1178
Marginal R^2 / Conditional R^2	0.486 / 0.531	0.452 / 0.498	0.361 / 0.457	0.436 / 0.501	0.334 / 0.363	0.397 / 0.470

* *p<0.05* ** *p<0.01* *** *p<0.001*

Linear mixed-effects models examining how self-reported transition likelihood (centered) influences estimated transition likelihood for others, with Loneliness (centered) and their interaction as predictors. Random effects include variance components at the participant, from-emotion, and to-emotion levels. Intraclass correlations (ICCs) indicate the proportion of variance attributable to these levels. The number of participants, from-emotion, and to-emotion levels, as well as total observations, are provided for each study. Marginal and conditional R^2 values represent the variance explained by fixed effects alone and by the full model, respectively. Significance levels: **p* < .05, **p < .01, ***p < .001.

Anchoring Results Controlling for Age

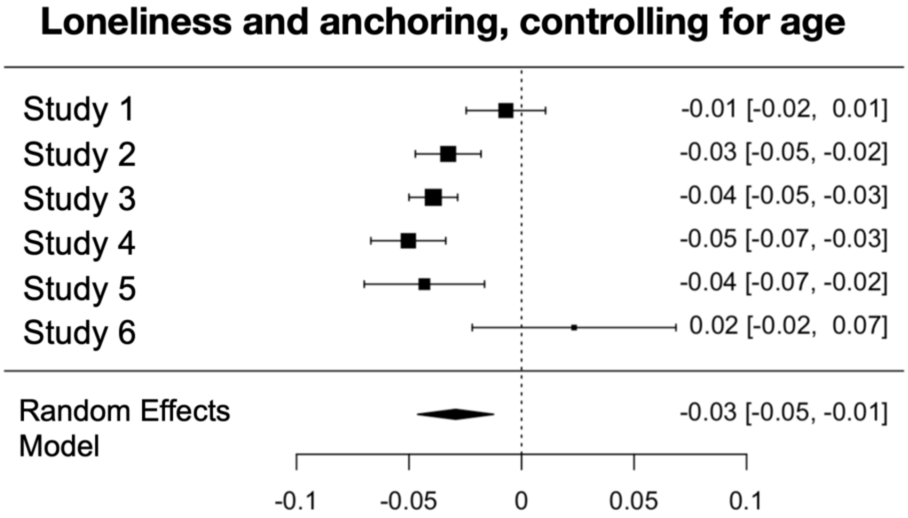

Supplementary Fig. 11

Meta-analytic Effects of the Interaction Between Anchoring and Loneliness, controlling for age

Effect size estimates of interaction between anchoring and loneliness, controlling for age, for each study for other ratings are visualized. Each square represents the correlation estimate from a single dataset with error bars denoting the corresponding 95% confidence intervals. The rhombus at the bottom represents the overall meta-analytic correlation estimate with its midpoint indicating the average effect size and its width representing the 95% confidence interval, summarizing the effect size across all included studies.

Loneliness and Volatility of Others' Emotion Transitions by Study

Supplementary Table 9

Loneliness and Estimated Transition Likelihood for Other Transitions by Valence

	Study 1	*Study 2*	*Study 3*	*Study 4*	*Study 5*	*Study 6*
Predictors	*Estimate (95% CI)*	*Estimate (95% CI)*	*Estimate (95% CI)*	*Estimate (95% CI)*	*Estimate (95% CI)*	*Estimate (95% CI)*
(Intercept)	65.821 *** (61.994 – 69.648)	68.268 *** (64.272 – 72.265)	70.147 *** (66.100 – 74.195)	68.397 *** (65.203 – 71.590)	66.526 *** (63.143 – 69.908)	76.634 *** (70.488 – 82.780)
Emotion Transition Valence [neg_pos]	-29.223 *** (-33.460 – -24.986)	-30.474 *** (-34.703 – -26.244)	-36.822 *** (-41.963 – -31.681)	-28.274 *** (-30.892 – -25.656)	-28.198 *** (-30.969 – -25.428)	-38.060 *** (-43.543 – -32.577)
Emotion Transition Valence [pos_neg]	-35.164 *** (-39.446 – -30.881)	-33.989 *** (-39.065 – -28.912)	-25.539 *** (-30.206 – -20.873)	-28.806 *** (-31.214 – -26.397)	-28.740 *** (-33.821 – -23.660)	-33.603 *** (-39.578 – -27.628)
Emotion Transition Valence [pos_pos]	11.845 *** (5.914 – 17.776)	10.503 ** (3.946 – 17.061)	-2.312 (-9.236 – 4.612)	7.444 *** (4.289 – 10.600)	8.110 ** (2.457 – 13.763)	-24.381 *** (-30.174 – -18.588)
Loneliness c	-0.185 * (-0.345 – -0.026)	-0.083 (-0.206 – 0.039)	-0.125 ** (-0.217 – -0.032)	-0.040 (-0.284 – 0.204)	0.000 (-0.161 – 0.162)	0.257 (-0.303 – 0.817)
Emotion Transition Valence [neg_pos] × Loneliness c	0.157 * (0.024 – 0.291)	0.173 ** (0.069 – 0.277)	0.276 *** (0.186 – 0.365)	0.461 *** (0.311 – 0.610)	0.046 (-0.145 – 0.238)	-0.453 (-0.977 – 0.072)
Emotion Transition Valence [pos_neg] × Loneliness c	0.224 ** (0.090 – 0.357)	0.237 *** (0.133 – 0.341)	0.213 *** (0.123 – 0.303)	0.373 *** (0.224 – 0.522)	0.088 (-0.104 – 0.279)	-0.222 (-0.747 – 0.304)
Emotion Transition Valence [pos_pos] × Loneliness c	-0.142 (-0.314 – 0.031)	-0.160 * (-0.295 – -0.026)	-0.142 * (-0.258 – -0.027)	-0.170 * (-0.313 – -0.028)	-0.265 * (-0.512 – -0.018)	-0.495 (-1.002 – 0.012)
**Random Effects**						
σ^2	523.54	504.49	408.85	410.31	449.92	508.88
τ_{00}	107.72 Participant	99.88 Participant	56.39 Participant	97.80 Participant	44.07 Participant	108.77 Participant
	5.20 fromEmotion	8.26 fromEmotion	7.28 fromEmotion	3.45 fromEmotion	9.52 fromEmotion	2.18 fromEmotion
	5.07 toEmotion	5.52 toEmotion	8.90 toEmotion	3.70 toEmotion	2.32 toEmotion	0.94 toEmotion
ICC	0.18	0.18	0.15	0.21	0.12	0.19
N	113 Participant	185 Participant	376 Participant	91 Participant	68 Participant	41 Participant
	6 fromEmotion	6 fromEmotion	6 fromEmotion	15 fromEmotion	6 fromEmotion	6 fromEmotion
	6 toEmotion	6 toEmotion	6 toEmotion	15 toEmotion	6 toEmotion	6 toEmotion

Observations	4068	6660	13533	6075	2556	1204
Marginal R^2 / Conditional R^2	0.330 / 0.454	0.331 / 0.454	0.342 / 0.441	0.353 / 0.489	0.320 / 0.376	0.176 / 0.297

** p<0.05 ** p<0.01 *** p<0.001*

Linear mixed-effects models predicting estimated transition likelihood for others as a function of emotion transition valence (reference level: negative-to-negative), loneliness (centered), and their interaction. Emotion transition valence categories include negative-to-positive, positive-to-negative, and positive-to-positive transitions. Random effects include variance components at the participant, from-emotion, and to-emotion levels. Intraclass correlations (ICCs) indicate the proportion of variance attributable to these levels. The number of participants, from-emotion, and to-emotion levels, as well as total observations, are provided for each study. Marginal and conditional R^2 values represent the variance explained by fixed effects alone and by the full model, respectively. Significance levels: *p < .05, **p < .01, ***p < .001.

Supplementary Table 10

Simple Slopes of Loneliness and Estimated Transition Likelihood for Others by Valence

**Transition Valence**	**Study 1 β (95% CI)**	**Study 2 β (95% CI)**	**Study 3 β (95% CI)**	**Study 4 β (95% CI)**	**Study 5 β (95% CI)**	**Study 6 β (95% CI)**
Positive to Negative	0.018 (-0.064, 0.099)	0.071* (0.008, 0.134)	0.033 (-0.007, 0.073)	0.130** (0.048, 0.212)	0.031 (-0.028, 0.091)	0.104 (-0.101., 0.309)
Negative to Positive	-0.013 (-0.094, 0.068)	0.041 (-0.022, 0.104)	0.056** (0.017, 0.096)	0.154*** (0.072, 0.236)	0.017 (-0.043, 0.076)	-0.059 (-0.263, 0.146)
Negative to Negative	-0.085* (-0.158, -0.012)	-0.038 (-0.095, 0.018)	-0.046** (-0.081, -0.012)	0.001 (-0.081, 0.084)	0.004 (-0.055, 0.064)	-0.030 (-0.249, 0.308)
Positive to Positive	-0.150** (-0.245, -0.055)	-0.113** (-0.187, -0.038)	-0.100*** (-0.148, -0.051)	-0.063 (-0.143, 0.018)	-0.111** (-0.171, -0.051)	-0.192 (-0.388, 0.003)

** p<0.05 ** p<0.01 *** p<0.001*

Results of simple slopes analyses examining the relationship between loneliness (standardized) and estimated transition likelihood for others across different emotion transition valence conditions (positive to positive, positive to negative, negative to positive, negative to negative) in each study. Standardized regression coefficients (β) and 95% confidence intervals are reported for each valence transition type.

Volatility of Emotion Transitions for Others by Study

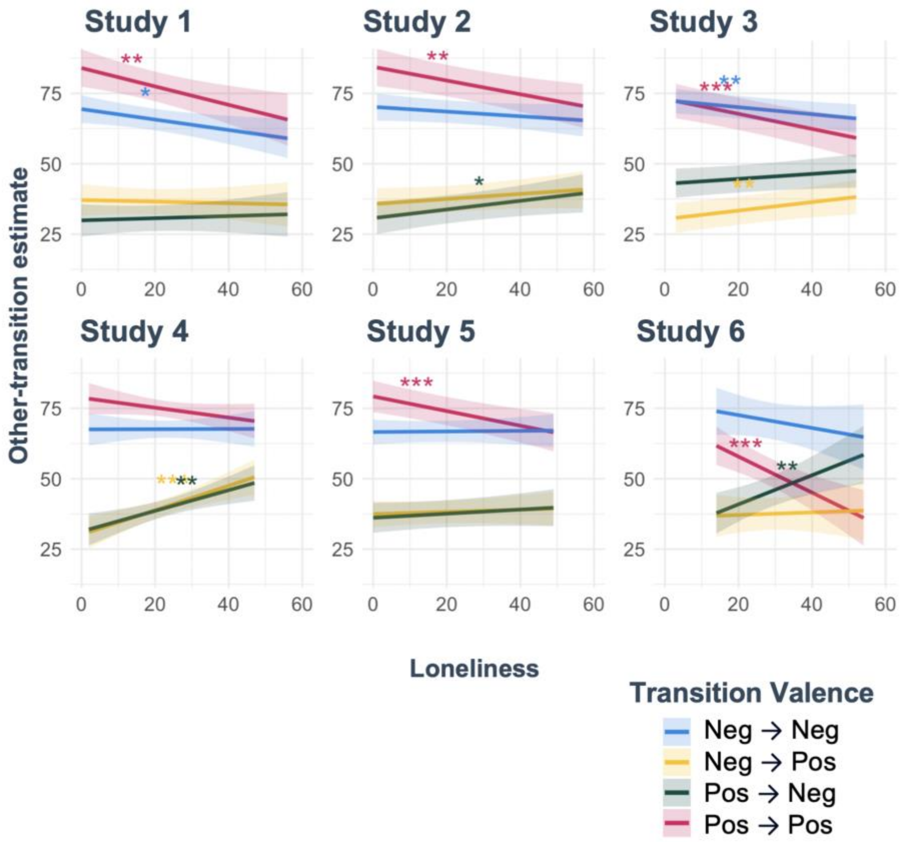

Supplementary Fig. 12

Simple Slopes for Each Valence Transition Type for Others' Transitions by Study

Simple slopes resulting from linear mixed-effects models for all datasets. Asterisks indicate p-values for simple slopes at each emotion transition valence type *** $p < .001$, **$p < .01$, *$p < .05$.

Additional test of Volatility: Transition distances using the 3d Mind Model

Our main results suggest that loneliness may be linked with more volatile perceptions of others' emotion transitions (see Fig. 4 in the main manuscript). To further test this idea, we operationalized volatility using an alternative approach. Drawing on the 3d Mind Model of mental states[3,4], we computed the distance between each emotion pair as the Euclidean distance in a three-dimensional space defined by rationality, social impact, and valence[3,4] based on coordinates from an existing dataset[3] (available at: https://osf.io/8mtxq/; file *pc166.csv*). Some emotions were not included in this original dataset (and thus could not be analyzed within this model), so all trials for which an exact match was unavailable were excluded from these analyses. Specifically, for Studies 1, 2, 3, and 5, the emotion "sluggish" was excluded, while for Study 4, the emotions "bold," "assertive," "confident," "grouchy," "unrestrained," "lively," "talkative," and "love" were excluded. No exclusions were necessary for Studies 6 and 7. For each trial, the coordinates corresponding to the emotion transitioned from and the emotion transitioned to were extracted from the existing dataset, and the Euclidean distance between these two sets of coordinates was computed to yield a measure of distance. This metric was then used to assess whether lonely individuals were more likely to expect emotion transitions for pairs of emotions with greater distance in the 3d Mind Model space (i.e., more volatile transitions). Separate mixed-effects models were fit for self ratings and for other ratings. For these analyses, the dependent variable was the likelihood rating of emotion transitions (either self or other), with the centered distance, loneliness, and their interaction as independent variables. Random intercepts were included in each model for participant, emotion state transitioned from, and emotion state transitioned to in order to account for nonindependence in our data from repeated observations for each participant and emotion. A significant interaction term here indicates that

loneliness moderates the strength of the relationship between transition distance and the predicted likelihood of transition (i.e., volatility of expectations). Study-specific estimates were aggregated using the same meta-analytic methods used throughout the main text.

In line with the findings in main text, we found a significant interaction between loneliness and transition distance in predicting likelihood of emotion transitions for others (β = 0.044, *SE* = 0.008, $p < .001$, 95% CI [0.028, 0.060]). These findings suggest that lonely individuals were more likely to perceive others to transition between emotion states further apart in transition. There was also a significant interaction between loneliness and the magnitude of emotional jumps, β = 0.045, *SE* = 0.016, $p = .004$, 95% CI [0.015, 0.076], such that lonely individuals showed a weaker negative association between distance and likelihood of transition (Supplementary Fig. 13 & 14). There was no significant main effect of loneliness on emotion transition likelihood (β = 0.009, *SE* = 0.015, $p = .552$, 95% CI [-0.020, 0.038]).

These findings suggest that lonely individuals perceive others as more volatile in general across this 3D space, expecting more dramatic shifts in emotional states as more likely compared to non-lonely individuals.

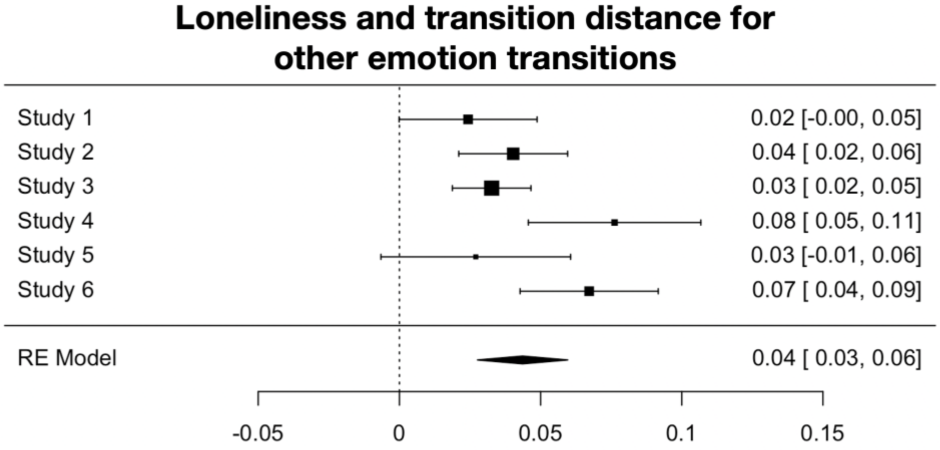

Supplementary Fig. 13
Estimates of Loneliness and Transition Distance for Other Ratings
Study specific estimates of the interaction effect of transition distance and loneliness for ratings of emotion transitions for other ratings. Each square represents the estimated effect (standardized) from a single dataset, with error bars indicating the corresponding 95% confidence intervals. The rhombus at the bottom represents the overall meta-analytic estimate, with its midpoint indicating the pooled effect size and its width representing the 95% confidence interval, summarizing the effect across all included studies.

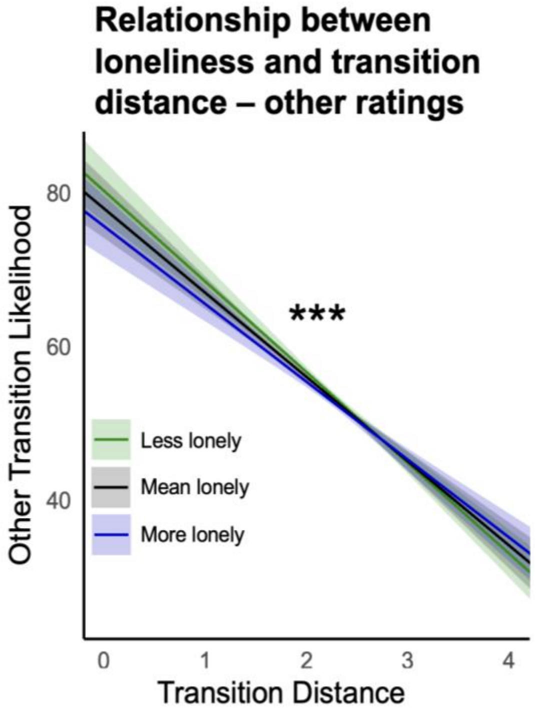

Supplementary Fig. 14
Relationship between Loneliness and Transition Distance for Other Ratings
The lines represent the predicted relationship between emotion transition ratings for others and transition distance at different levels of loneliness. Lower loneliness in green (-1 SD), average in black (mean), and higher in blue (+1 SD). The shaded areas indicate the 95% confidence intervals. This significant interaction suggests that loneliness moderates the extent to which distance is negatively associated with transition likelihood estimates for others. Asterisks indicate significance level: $p < 0.01$ (**).

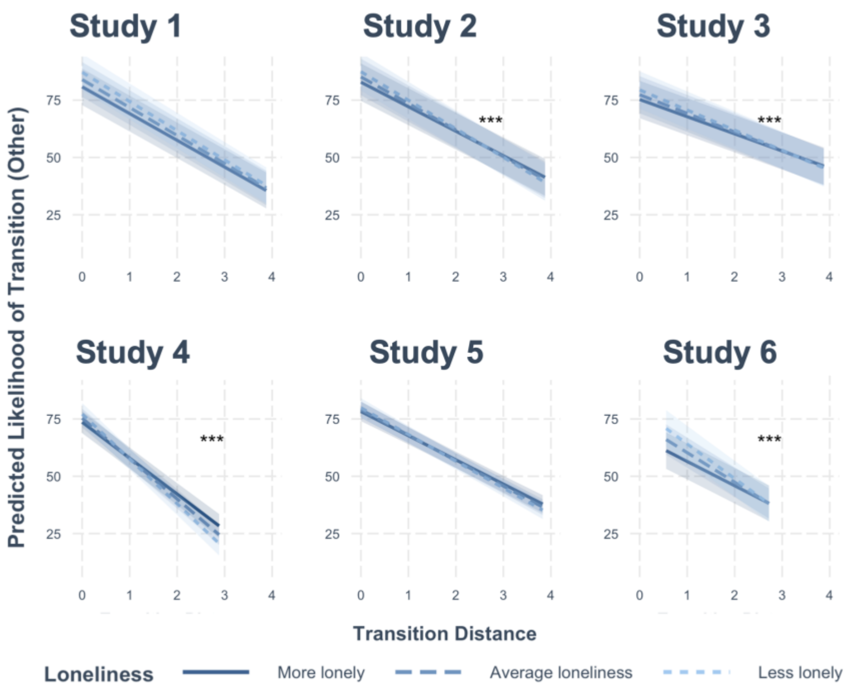

Supplementary Fig. 15

Relationship between Transition Distance and Loneliness for Individual Studies

Linear mixed-effects models for all datasets. p values for interactions between estimated likelihood of other's emotion transition and loneliness are indicated as follows: *** $p < .001$, **$p < .01$, *$p < .05$.

Analyses of individual dimensions of 3D model

To identify whether the observed volatility effect is driven by a particular dimension of the 3d Mind Model (i.e., valence, social impact, and/or rationality), we also calculated the distance between emotion states on each dimension. Specifically, we subtracted the location in one dimension of the *state transitioned from* from *the state transitioned to*. This distance measure allowed us to test whether the transition moved to was *higher* on that dimension (positive number) or *lower* (negative number). The quadratic of this term in turn allowed us to examine the absolute distance between states. We first examined the effect on ratings of others using dimensional distance, the quadratic of distance, loneliness, the interaction between loneliness and distance, and the interaction between loneliness and quadratic distance as fixed effects. Again, we fit random intercepts for participant, emotion item transitioned from, and emotion item transitioned to. For all these analyses of individual dimensions, study-specific estimates were aggregated using the same meta-analytic methods used throughout the main text.

Valence

Our analysis investigating the valence dimension showed a nonsignificant overall linear interaction effect between loneliness and valence distance, $\beta = -0.0214$, $SE = 0.0193$, $p = .268$, 95% CI [-0.059,0.017] (Supplementary Fig. 16A). However, there was a significant quadratic interaction between loneliness and valence distance^2, $\beta = 0.0368$, $SE = 0.0086$, $p < .001$, 95% CI [0.020, 0.054] (Supplementary Fig. 16B).

Given these significant interactions, we ran post-hoc tests to examine the marginal estimates of the relationship between loneliness and ratings at different levels of valence transitions (Supplementary Fig. 17). For transitions involving the largest negative shift, a random-effects model indicated a statistically significant overall effect, $\beta = 0.072$, $SE = 0.015$, p

< .001, 95% CI [0.043, 0.101]. This suggests that loneliness was associated with an increased likelihood of rating others as undergoing a substantial change toward a negative state. For transitions with no valence change, a random-effects model revealed a statistically significant overall effect, β = -0.051, *SE* = 0.018, *p* = .006, 95% CI [-0.087, -0.015]. In other words, as loneliness increased, participants were less likely to expect stable transitions, whereas less lonely participants were more likely to anticipate no change in valence. For transitions involving the largest positive shift, a random-effects model showed a marginally significant overall effect, β = 0.045, *SE* = 0.026, *p* = .09, 95% CI [-0.007, 0.097]. Although this finding did not reach conventional levels of significance, it indicates that higher loneliness was marginally associated with an increased likelihood of rating others as transitioning to more positive states. Overall, these results suggest that lonely individuals were more likely to expect others to undergo substantial negative shifts and also marginally expected others to undergo large positive shifts. Correspondingly, non-lonely participants tended to anticipate stable outcomes with no change in valence. This is in line with our findings in main text that lonely individuals expect more volatile valence shifts compared to non-lonely individuals.

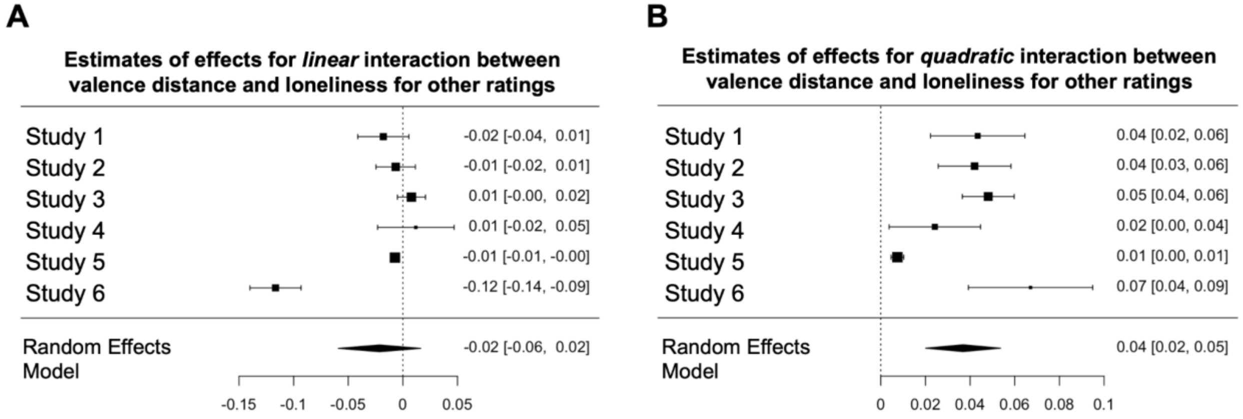

Supplementary Fig. 16

Estimates for Interaction between Valence Distance and Loneliness for Other Ratings
(A) Study estimates contributing to the meta-analysis of the linear interaction effect of valence distance and loneliness for ratings of emotion transitions for other. The rhombus at the bottom represents the overall meta-analytic estimate, which is non-significant. **(B)** Study estimates contributing to the meta-analysis of the *quadratic* interaction effect of valence distance and loneliness for ratings of other's emotion transitions. The rhombus at the bottom represents the overall meta-analytic estimate, which is significant at $p < .001$

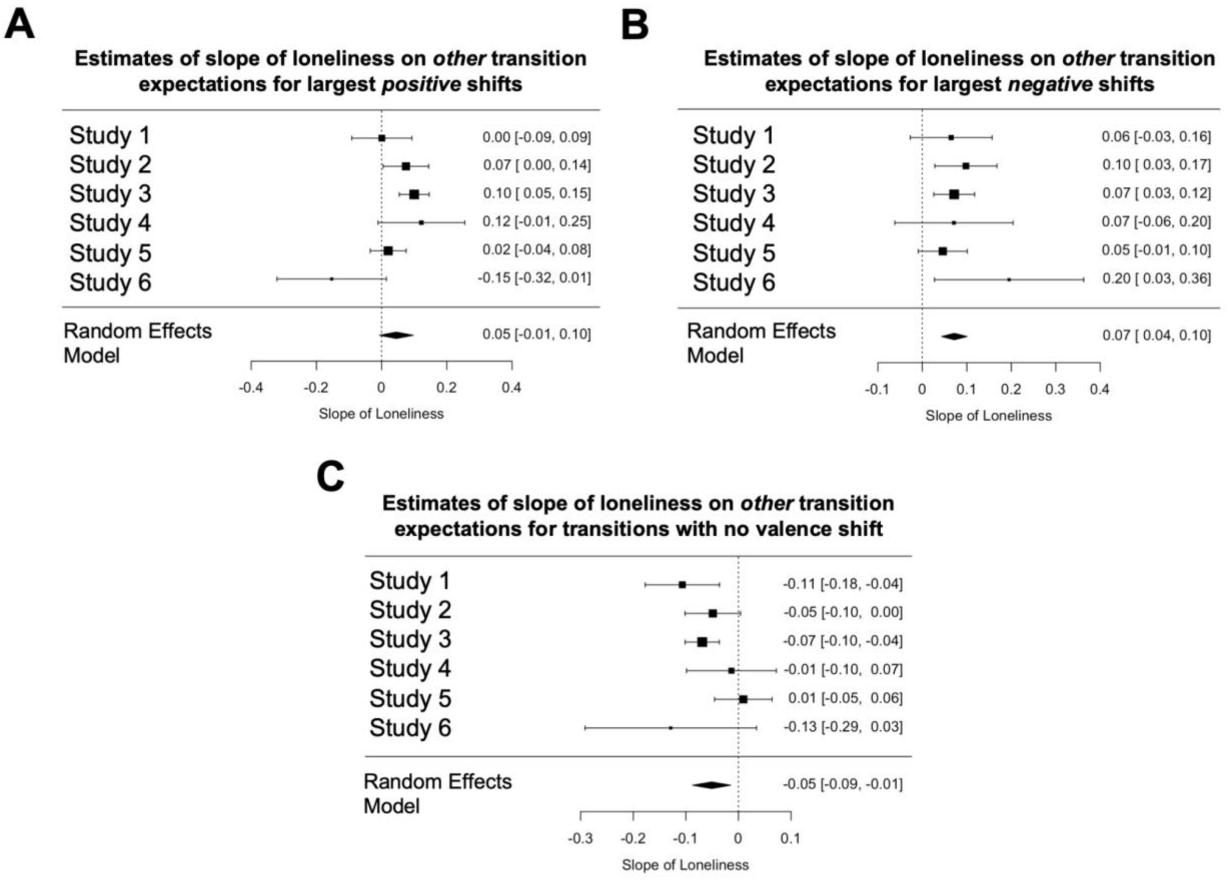

Supplementary Fig. 17
Slope estimates at different valence transitions for other
(A) Slope estimates from each study of the association between loneliness and ratings of others' transition likelihoods for transitions with the largest positive valence shifts. Loneliness was marginally positively associated with these transition likelihood ratings. **(B)** Slope estimates from each study for transitions with the largest negative valence shifts. Loneliness was positively associated with these transition likelihood ratings. **(C)** Slope estimates from each study for transitions with no valence shift. Loneliness was negatively associated with these transition likelihood ratings, such that less lonely individuals rated same-valence transitions as more likely. Each square represents the estimated interaction effect (standardized) from a single dataset, with error bars indicating the corresponding 95% confidence intervals. The rhombus at the bottom represents the overall meta-analytic estimate.

Social Impact & Rationality

We repeated the above analyses for the social impact dimension and fit a mixed-effects model with emotion transition ratings for others as the dependent variable and the social impact transition distance, the quadratic term for social impact transition distance (to measure absolute difference), loneliness, the linear interaction between loneliness and social impact transition distance, and the quadratic interaction between loneliness and social impact transition distance (squared) as fixed effects. For the rationality dimension, we fit an analogous mixed-effects model with emotion transition ratings for others the dependent variable and rationality transition distance, the quadratic term for rationality transition distance (to measure absolute difference), loneliness, the linear interaction between loneliness and rationality transition distance, and the quadratic interaction between loneliness and rationality transition distance (squared) as fixed effects. Both models included the same random effects as outlined above.

Our analysis investigating the social impact dimension showed a nonsignificant overall linear interaction effect between loneliness and social impact distance, $\beta = -0.012$, $SE = 0.007$, $p = .108$, 95% CI [-0.026, 0.026] (Supplementary Fig. 18A), as well as no significant quadratic interaction between loneliness and social impact distance^2, $\beta = 0.011$, $SE = 0.010$, $p = .284$, 95% CI [-0.009, 0.031] (Supplementary Fig. 18B). Our analysis investigating the rationality dimension also found no overall linear interaction effect between loneliness and rationality distance, $\beta = 0.002$, $SE = 0.002$, $p = .271$, 95% CI [-0.002, 0.006] (Supplementary Fig. 19A), and no significant quadratic interaction between loneliness and rationality distance^2, $\beta = 0.012$, $SE = 0.009$, $p = .158$, 95% CI [-0.005, 0.029] (Supplementary Fig. 19B). Given that we did not find significant interaction effects, we did not run post-hoc analyses of marginal estimates.

These results suggest that lonely individuals' perceptions of volatility for others are driven by the dimension of valence, corroborating our findings in the main manuscript.

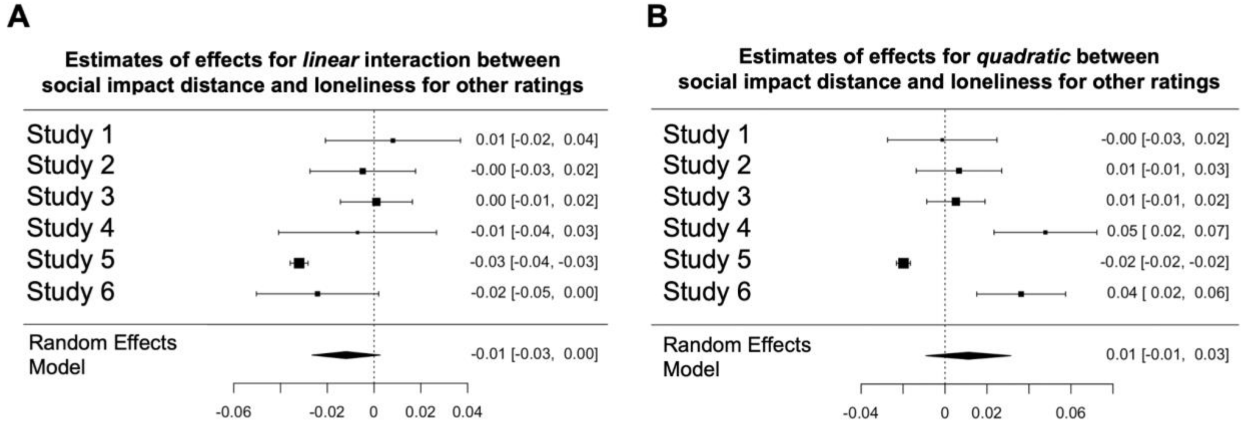

Supplementary Fig. 18
Estimates for Interaction between Social Impact Distance and Loneliness for Other Ratings
(A) Study estimates of the linear interaction effect of social impact distance and loneliness for ratings of emotion transitions for other. The rhombus at the bottom represents the overall meta-analytic estimate, which is non-significant. **(B)** Study estimates of the *quadratic* interaction effect of social impact distance and loneliness for ratings of other's emotion transitions. The rhombus at the bottom represents the overall meta-analytic estimate, which is non-significant. Each square represents the estimated interaction effect (standardized) from a single dataset, with error bars indicating the corresponding 95% confidence intervals.

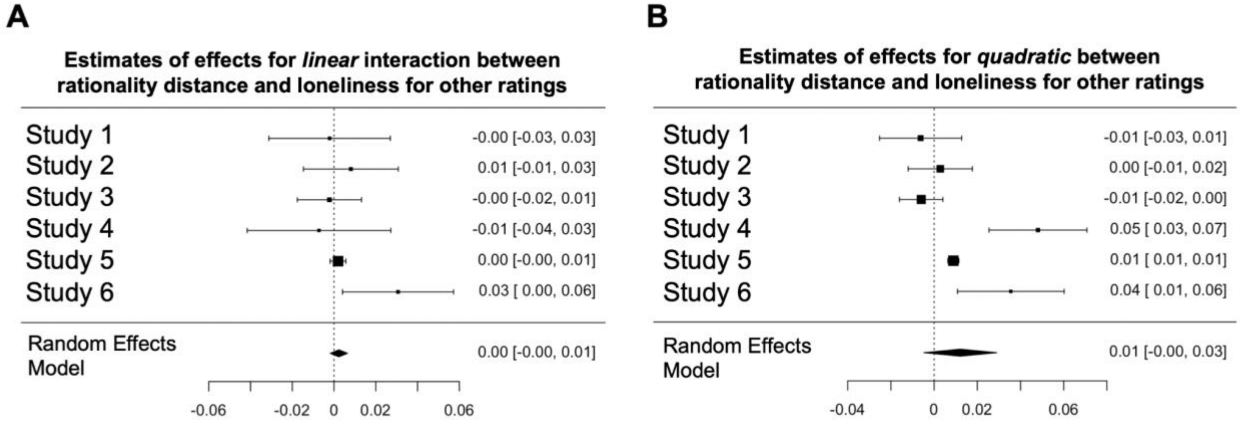

Supplementary Fig. 19
Estimates for Interaction between Rationality Distance and Loneliness for Other Ratings
(A) Study estimates contributing of the linear interaction effect of rationality distance and loneliness for ratings of emotion transitions for other. The rhombus at the bottom represents the overall meta-analytic estimate, which is non-significant. **(B)** Study estimates of the *quadratic* interaction effect of rationality distance and loneliness for ratings of other's emotion transitions. The rhombus at the bottom represents the overall meta-analytic estimate, which is non-significant. Each square represents the estimated interaction effect (standardized) from a single dataset, with error bars indicating the corresponding 95% confidence intervals.

Loneliness and Confidence for Others Ratings

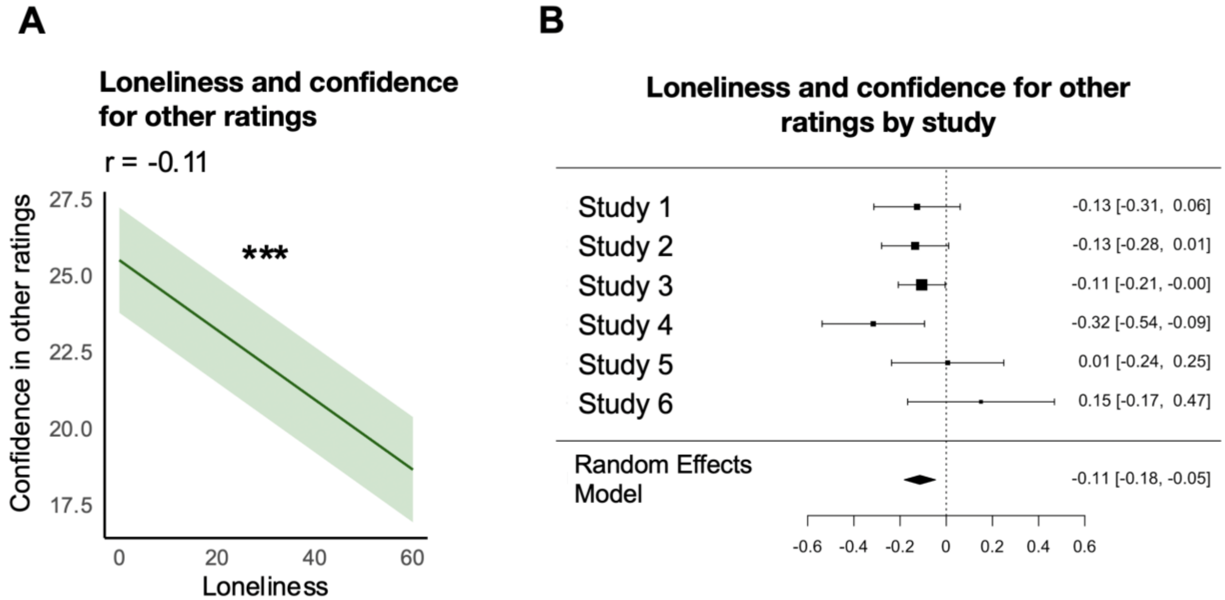

Supplementary Fig. 20
Loneliness is associated with decreased confidence in ratings of others' emotion transitions
(A) Loneliness was negatively associated with confidence in emotion transition predictions for others. The dark green line represents the meta-analytic correlation estimate, while the light green bands indicate the 95% confidence interval. Higher loneliness was associated with lower by-participant standard deviations, suggesting that lonely participants provided less variable ratings. Asterisks indicate significance level: $p < .001$ (***). **(B)** Correlation estimates from individual datasets are visualized. Each square represents the correlation estimate from a single dataset, with error bars denoting the corresponding 95% confidence intervals. The rhombus at the bottom represents the overall meta-analytic correlation estimate with its midpoint indicating the average effect size and its width representing the 95% confidence interval, summarizing the effect size across all included studies.

Loneliness and Confidence for Others Ratings: By Study

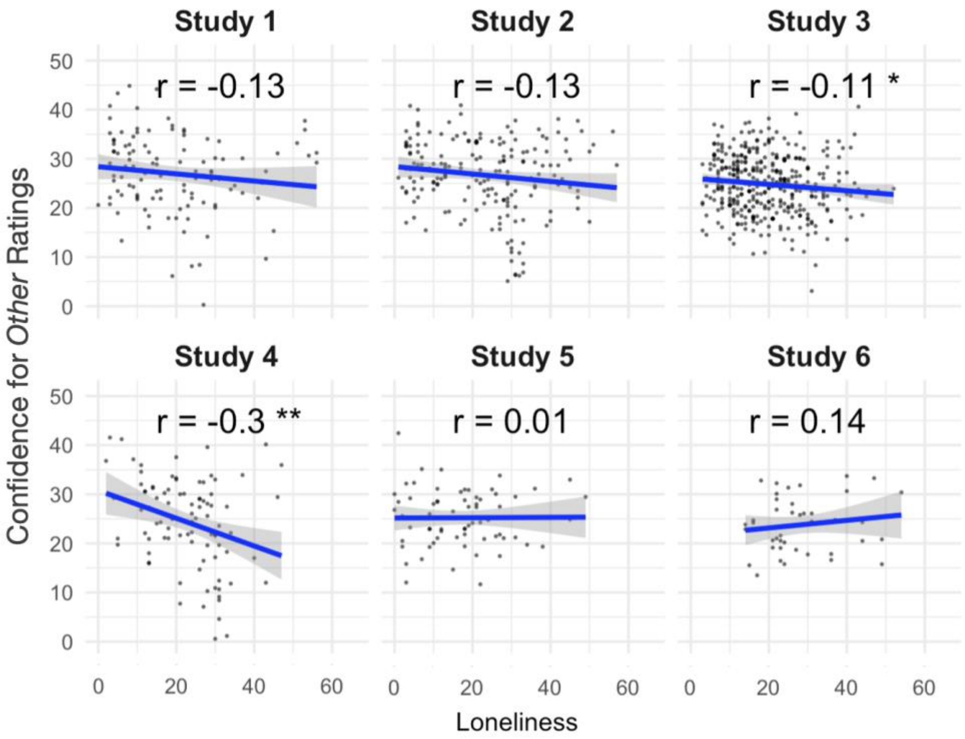

Supplementary Fig. 21
Correlations of loneliness and confidence for ratings for others
Pearson correlations for the relationship between SD by participant and loneliness for other ratings. *p* values are indicated as follows: *** $p < .001$, **$p < .01$, *$p < .05$.

Volatility of Self Emotion Transitions by Study

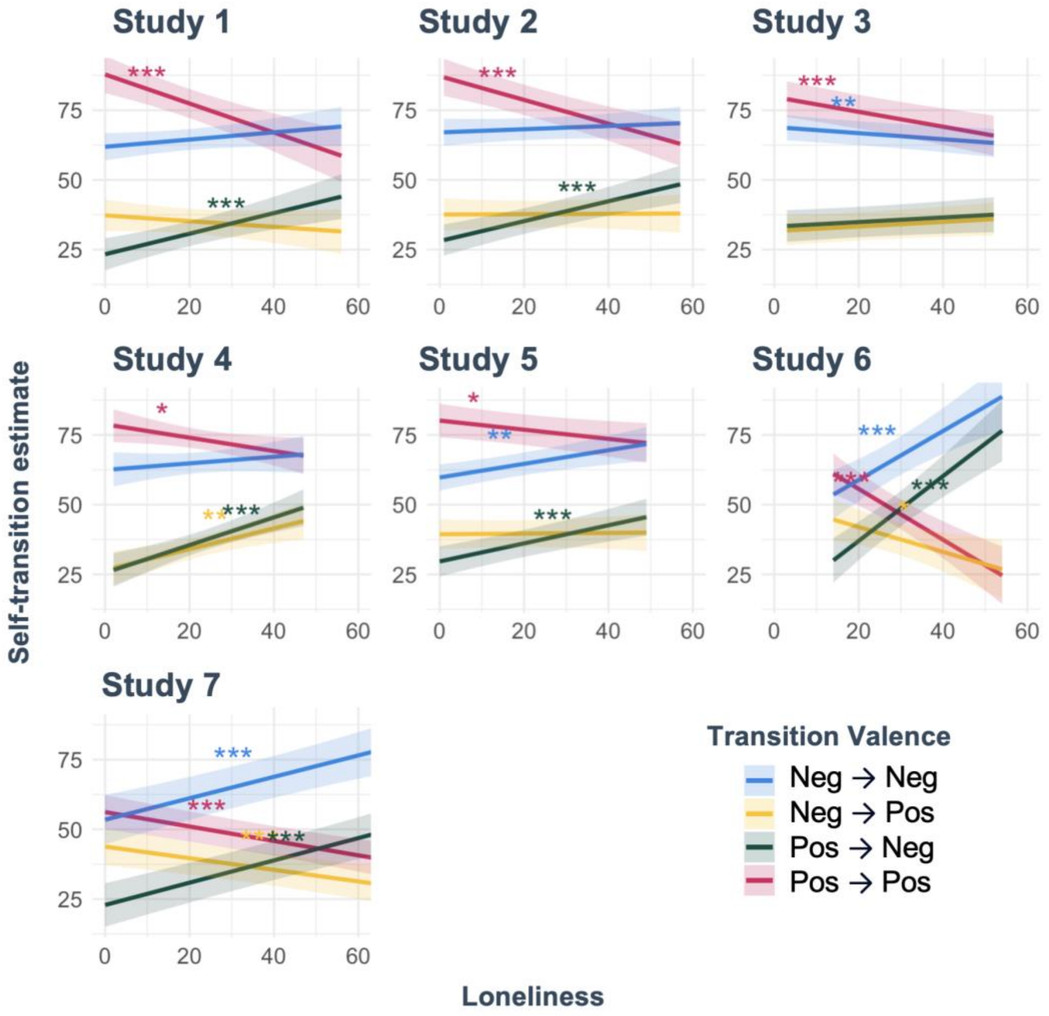

Supplementary Fig. 22

By Study Simple Slopes for Each Valence Transition Type for Self Transitions

Simple slopes resulting from linear mixed-effects models for all datasets. Asterisks indicate p-values for simple slopes at each emotion transition valence type *** $p < .001$, **$p < .01$, *$p < .05$.

Supplementary Table 11

Loneliness and Estimated Transition Likelihood for Self by Valence

	Study 1	*Study 2*	*Study 3*	*Study 4*	*Study 5*	*Study 6*	*Study 7*
Predictors	*Estimate (95% CI)*	*Estimate (95% CI)*	*Estimate (95% CI)*	*Estimate (95% CI)*	*Estimate (95% CI)*	*Estimate (95% CI)*	*Estimate (95% CI)*
(Intercept)	64.409 *** (60.634 – 68.184)	68.381 *** (64.480 – 72.282)	66.827 *** (62.526 – 71.127)	65.153 *** (61.439 – 68.866)	64.004 *** (59.964 – 68.044)	71.305 *** (63.452 – 79.158)	64.188 *** (56.413 – 71.964)
Emotion Transition Valence [neg_pos]	-29.102 *** (-33.264 – -24.940)	-30.632 *** (-35.512 – -25.753)	-33.534 *** (-38.138 – -28.930)	-29.982 *** (-33.021 – -26.943)	-24.416 *** (-28.883 – -19.949)	-32.952 *** (-40.335 – -25.568)	-26.557 *** (-35.046 – -18.068)
Emotion Transition Valence [pos_neg]	-33.894 *** (-38.127 – -29.661)	-32.003 *** (-36.110 – -27.896)	-31.956 *** (-37.774 – -26.137)	-28.153 *** (-31.657 – -24.649)	-28.714 *** (-33.348 – -24.081)	-27.856 *** (-34.528 – -21.184)	-28.002 *** (-32.344 – -23.660)
Emotion Transition Valence [pos_pos]	13.321 *** (7.489 – 19.153)	8.951 ** (2.632 – 15.271)	7.710 * (0.308 – 15.113)	8.131 *** (3.830 – 12.432)	13.281 *** (6.846 – 19.716)	-21.967 *** (-30.266 – -13.667)	-16.874 *** (-26.308 – -7.440)
Loneliness c	0.129 (-0.033 – 0.292)	0.058 (-0.074 – 0.189)	-0.110 ** (-0.191 – -0.028)	0.118 (-0.112 – 0.348)	0.245 *** (0.104 – 0.385)	0.497 (-0.036 – 1.031)	0.436 *** (0.316 – 0.556)
Emotion Transition Valence [neg_pos] × Loneliness c	-0.232 ** (-0.372 – -0.092)	-0.052 (-0.162 – 0.059)	0.192 *** (0.103 – 0.280)	0.256 ** (0.098 – 0.413)	-0.232 *** (-0.253 – -0.212)	-0.818 *** (-1.299 – -0.338)	-0.617 *** (-0.724 – -0.509)
Emotion Transition Valence [pos_neg] × Loneliness c	0.239 *** (0.099 – 0.380)	0.299 *** (0.189 – 0.410)	0.192 *** (0.103 – 0.281)	0.379 *** (0.221 – 0.536)	0.079 *** (0.059 – 0.100)	0.238 (-0.242 – 0.718)	-0.050 (-0.158 – 0.057)
Emotion Transition Valence [pos_pos] × Loneliness c	-0.650 *** (-0.831 – -0.469)	-0.483 *** (-0.626 – -0.341)	-0.157 ** (-0.272 – -0.043)	-0.357 *** (-0.507 – -0.207)	-0.409 *** (-0.435 – -0.382)	-1.138 *** (-1.602 – -0.673)	-0.668 *** (-0.772 – -0.564)
**Random Effects**							
σ^2	577.36	570.86	399.28	488.26	527.12	542.44	640.39
τ_{00}	109.71 Participant	115.50 Participant	38.82 Participant	91.04 Participant	44.70 Participant	114.19 Participant	179.68 Participant
	4.94 fromEmotion	5.08 fromEmotion	11.48 fromEmotion	9.41 fromEmotion	7.43 fromEmotion	5.65 fromEmotion	5.92 fromEmotion
	4.73 toEmotion	7.49 toEmotion	7.09 toEmotion	6.44 toEmotion	6.91 toEmotion	9.12 toEmotion	24.39 toEmotion
ICC	0.17	0.18	0.13	0.18	0.10	0.19	0.25
N	113 Participant	185 Participant	376 Participant	91 Participant	68 Participant	41 Participant	856 Participant
	6 fromEmotion	6 fromEmotion	6 fromEmotion	15 fromEmotion	6 fromEmotion	6 fromEmotion	6 fromEmotion
	6 toEmotion	6 toEmotion	6 toEmotion	15 toEmotion	6 toEmotion	6 toEmotion	6 toEmotion
Observations	4068	6660	13535	6075	2556	1200	25618
Marginal R^2 / Conditional R^2	0.315 / 0.432	0.291 / 0.421	0.394 / 0.471	0.349 / 0.465	0.290 / 0.340	0.117 / 0.279	0.076 / 0.304

* *p<0.05* ** *p<0.01* *** *p<0.001*

Linear mixed-effects models predicting estimated transition likelihood for self as a function of emotion transition valence (reference level: negative-to-negative), loneliness (centered), and their interaction. Emotion transition valence categories include negative-to-positive, positive-to-negative, and positive-to-positive transitions. Random effects include variance components at the participant, from-emotion, and to-emotion levels. Intraclass correlations (ICCs) indicate the proportion of variance attributable to these levels. The number of participants, from-emotion, and to-emotion levels, as well as total observations, are provided for each study. Marginal and conditional R^2 values represent the variance explained by fixed effects alone and by the full model, respectively. Significance levels: **p* < .05, **p < .01, ****p* < .001.

Supplementary Table 12

Simple Slopes of Loneliness and Estimated Transition Likelihood for Self by Valence

Transition Valence	Study 1 β (95% CI)	Study 2 β (95% CI)	Study 3 β (95% CI)	Study 4 β (95% CI)	Study 5 β (95% CI)	Study 6 β (95% CI)	Study 7 β (95% CI)
Positive to Negative	0.164*** (0.083, 0.245)	0.158*** (0.094, 0.223)	0.030 (-0.005, 0.066)	0.164*** (0.089, 0.240)	0.131*** (0.074, 0.187)	0.374*** (0.174, 0.572)	0.160** (0.124, 0.196)
Negative to Positive	-0.046 (-0.127, 0.035)	0.003 (-0.062, 0.067)	0.030 (-0.005, 0.066)	0.123** (0.048, 0.199)	0.005 (-0.052, 0.062)	-0.163 (-0.362, 0.036)	-0.075*** (-0.111, -0.039)
Negative to Negative	0.058 (-0.015, 0.130)	0.026 (-0.033, 0.084)	-0.041** (-0.071, -0.010)	0.039 (-0.037, 0.115)	0.099** (0.042, 0.155)	0.253 (-0.019, 0.525)	0.180*** (0.131, 0.230)
Positive to Positive	-0.232*** (-0.327, -0.136)	-0.189*** (-0.265, -0.113)	-0.099*** (-0.143, -0.054)	-0.079* (-0.153, -0.005)	-0.066* (-0.123, -0.009)	-0.325*** (-0.514, -0.136)	-0.096*** (-0.130, -0.062)

* *p<0.05* ** *p<0.01* *** *p<0.001*

Results of simple slopes analyses examining the relationship between loneliness (standardized) and estimated transition likelihood for self across different emotion transition valence conditions (positive to positive, positive to negative, negative to positive, negative to negative) in each study. Standardized regression coefficients (β) and 95% confidence intervals are reported for each valence transition type.

Transition Distance - Self

We repeated our analyses for transition distances of the overall 3d Mind Model[1,2] for ratings for emotion transitions for the self. We again used the Euclidean distance between the coordinates for the emotions transitioned to and from to yield a measure of distance. This metric was then used to assess whether lonely individuals were more likely to expect emotion transitions for pairs of emotions with greater distance in the 3d Mind Model space (i.e., more volatile transitions). We fit a mixed effect model with the likelihood rating of self emotion transitions as the dependent variable, and centered distance, loneliness, and their interaction as independent variables. Random intercepts were included in each model for participant, emotion state transitioned from, and emotion state transitioned to in order to account for nonindependence in our data from repeated observations for each participant and emotion. A significant interaction term here indicates that loneliness moderates the strength of the relationship between transition distance and predicted likelihood of transition (i.e., volatility of expectations). Study-specific estimates were aggregated using the same meta-analytic methods used throughout the main text.

We found a negative main effect of transition distance, $\beta = -0.504$, $SE = 0.041$, $p < .001$, 95% CI [-0.585, -0.424], such that individuals generally expected themselves to be less likely to transition between states that were more distant in 3D space. There was also a significant interaction between loneliness and the magnitude of emotional jumps, $\beta = 0.030$, $SE = 0.006$, $p < .001$, 95% CI [0.018, 0.042], such that lonelier individuals showed a weaker negative association between distance and likelihood of transition (Supplemental Figures 23 & 24). There was no significant main effect of loneliness, $\beta = 0.009$, $SE = 0.015$, $p = .552$, 95% CI [-0.020, 0.038].

These findings suggest that lonely individuals also viewed themselves as more volatile in general across this 3D space, seeing more dramatic shifts in emotional states as more likely compared to non-lonely individuals.

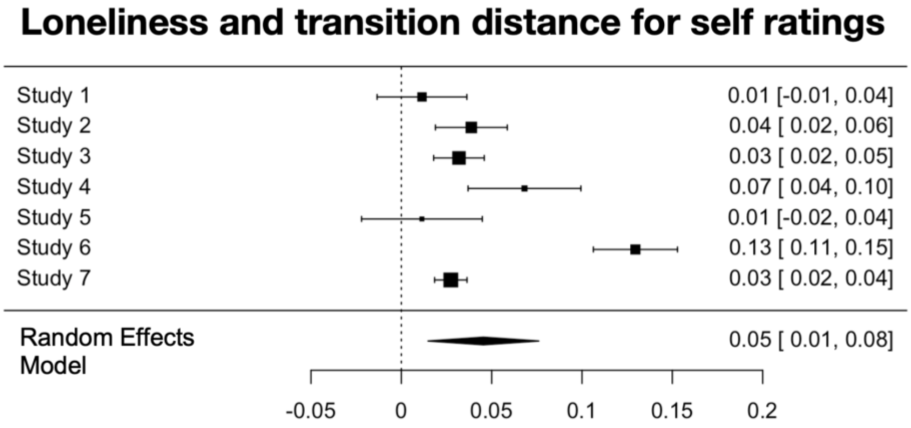

Supplementary Fig. 23

Estimates for Interaction between Transition Distance and Loneliness for Self Ratings

Study estimates of the interaction effect of transition distance and loneliness for ratings of self emotion transitions. Each square represents the estimated interaction effect (standardized) from a single dataset, with error bars indicating the corresponding 95% confidence intervals. The rhombus at the bottom represents the overall meta-analytic estimate, with its midpoint indicating the pooled effect size and its width representing the 95% confidence interval, summarizing the effect across all included studies.

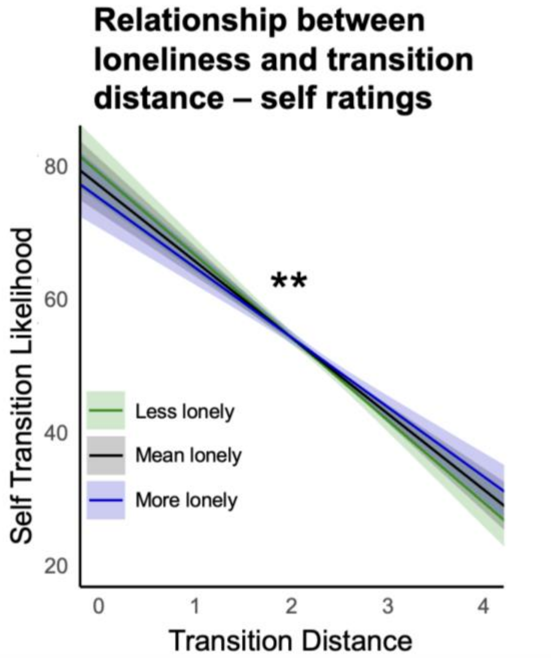

Supplementary Fig. 24

Relationship between Loneliness and Transition Distance for Self Ratings

The lines represent the predicted relationship between emotion transition ratings for the self and transition distance at different levels of loneliness. Lower loneliness in green (-1 SD), average in black (mean), and higher in blue (+1 SD). The shaded areas indicate the 95% confidence intervals. This significant interaction suggests that predicted likelihood of transition falls off less sharply with distance in lonelier individuals. Asterisks indicate significance level: $p < 0.01$ (**).

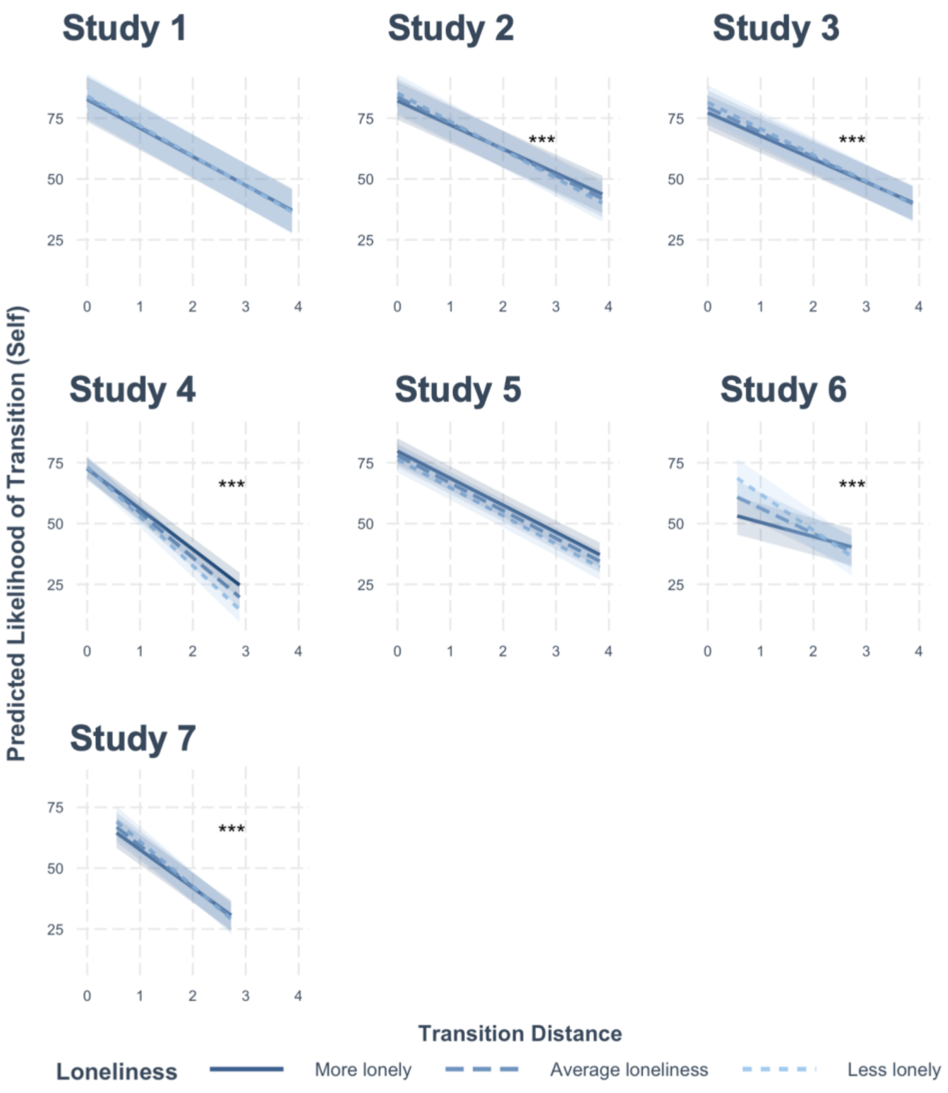

Supplementary Fig. 25

Relationship between Transition Distance and Loneliness for Individual Studies (self)

Linear mixed-effects models for all datasets. *p* values for interactions between estimated likelihood of self emotion transition and loneliness are indicated as follows: *** $p < .001$, ** $p < .01$, * $p < .05$.

Valence

To better understand the observed volatility characterizing loneliness and given our finding that lonely individuals perceive themselves to be less likely to persist in positive states, we next sought to understand valence differences using the 3d Mind Model[3]. We fit mixed-effects models with emotion transition ratings for the self as the dependent variable and valence transition distance, the quadratic term for valence transition distance (to measure absolute difference), loneliness, and the linear interaction between loneliness and valence transition distance, as well as the quadratic interaction between loneliness and valence transition distance (squared) as fixed effects.

We found a significant overall linear interaction effect between loneliness and valence distance, $\beta = -0.065$, $SE = 0.025$, $p = .009$, 95% CI [-0.113,-0.016] (Supplementary Fig. 26A), as well as a significant quadratic interaction between loneliness and valence distance^2, $\beta = 0.028$ $SE = 0.007$, $p < .001$, 95% CI [0.014, 0.043] (Supplementary Fig. 26B).

Given these significant interactions, we conducted post-hoc analyses to estimate the marginal effects of loneliness on self ratings at different levels of valence distance (Supplementary Fig. 27). For transitions involving the largest negative shift, a random-effects model indicated a statistically significant overall effect, $\beta = 0.162$, $SE = 0.027$, $p < .0001$, 95% CI [0.109, 0.214]. This suggests that higher loneliness was associated with an increased likelihood of expecting themselves to undergo a substantial change toward a negative state. This is in line with our findings in the main text, suggesting that lonely individuals were likely to move away from positive states and into negative ones. For transitions with no valence change, a random-effects model revealed a statistically nonsignificant overall effect, $\beta = -0.017$, $SE = 0.019$, $p = 0.386$, 95% CI [-0.055, 0.021]. In other words, for the self, there was no association

between loneliness and likelihood of transitioning to a similar-valence state. For transitions involving the largest positive shift, a random-effects model showed a nonsignificant overall effect, β = -0.023, *SE* = 0.047, *p* = 0.617, 95% CI [-0.115, 0.068]. In other words, there was no association between loneliness and likelihood of transitioning into more positive states, in line with our categorical findings described in main text. Overall, these results suggest that lonely individuals were more likely to expect themselves to move into more negative states. This is in line with our findings in main text that lonely individuals see positive states as more fragile. These results further suggest that more negative states are an attractor state for lonely individuals.

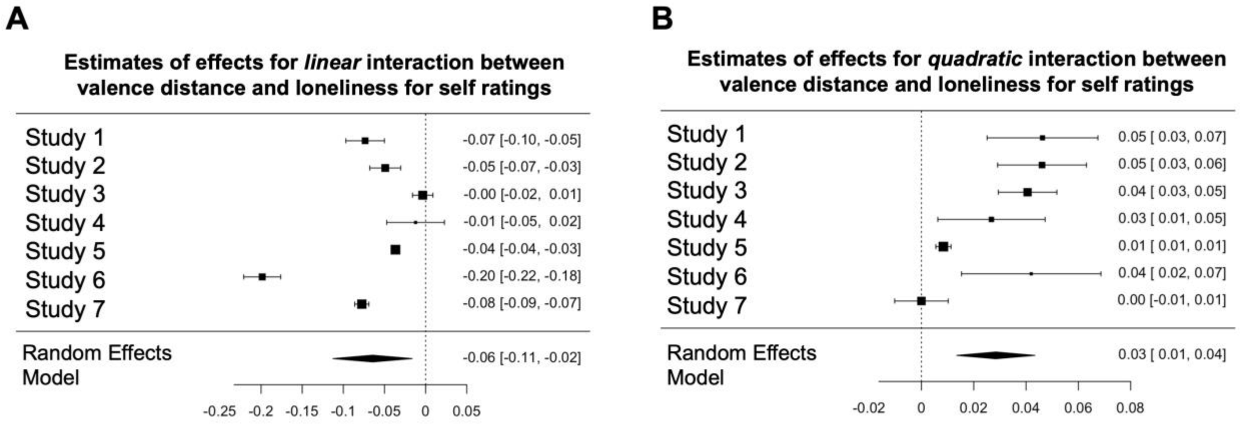

Supplementary Fig. 26
Estimates for Interaction between Valence Distance and Loneliness for Self Ratings
(A) Study estimates of the linear interaction effect of valence distance and loneliness for ratings of emotion transitions for self. **(B)** Study estimates of the *quadratic* interaction effect of valence distance and loneliness for ratings of self emotion transitions. The rhombus at the bottom represents the overall meta-analytic estimate, which is significant at $p < .001$

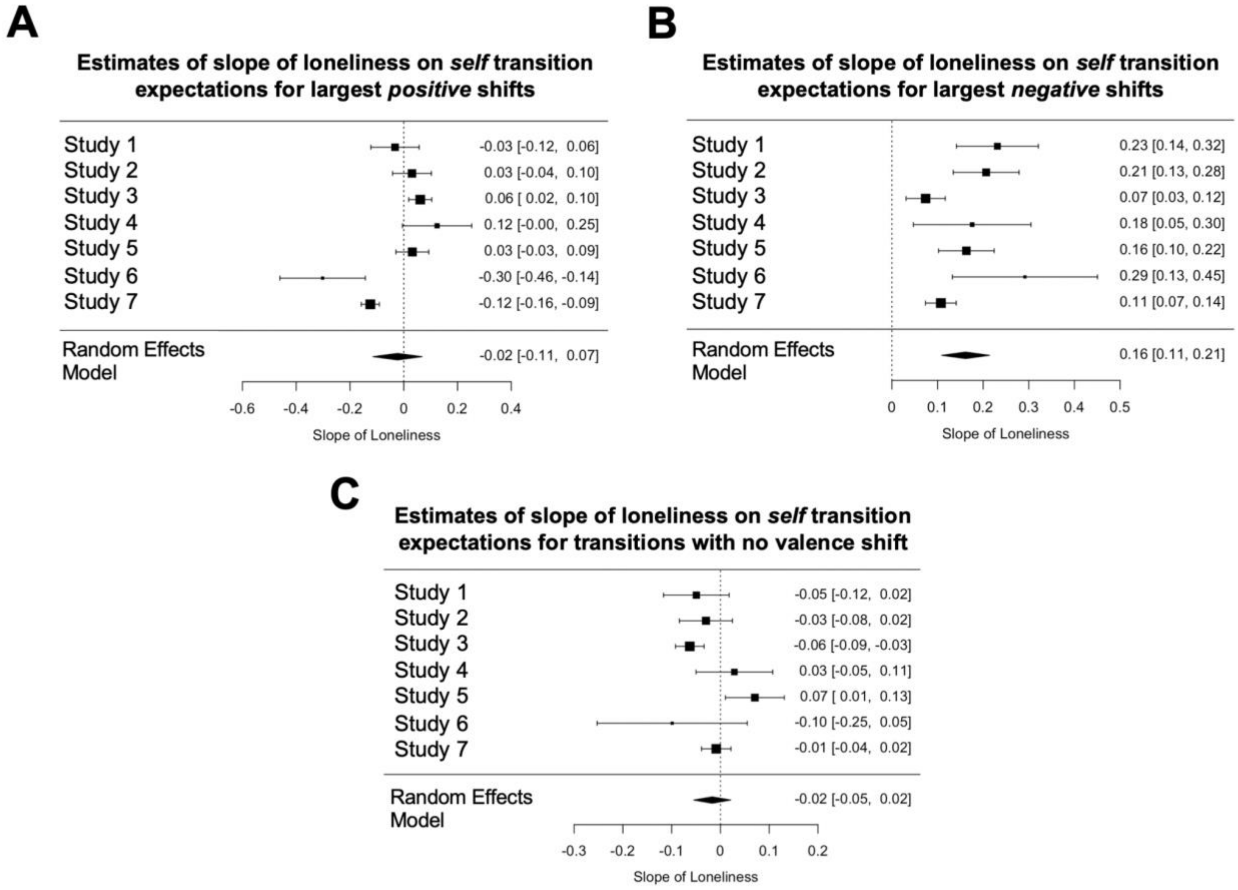

Supplementary Fig. 27

Slope estimates at different valence transitions for self

(A) Slope estimates by study of the slope of loneliness on ratings of self transition likelihoods at transitions with the largest positive shifts. No significant association was found for loneliness at these positive shifts. **(B)** Slope estimates by study of the slope of loneliness on ratings of self transition likelihoods at transitions with the largest negative shifts. Loneliness was positively associated with transition likelihood at these negative shifts. **(C)** Slope estimates by study of the slope of loneliness on ratings of self transition likelihoods at transitions with no valence shift. No significant negative association was found for loneliness at these neutral shifts. Each square represents the estimated interaction effect (standardized) from a single dataset, with error bars indicating the corresponding 95% confidence intervals. The rhombus at the bottom represents the overall meta-analytic estimate.

Social impact & Rationality

For completeness, as in our analyses of expectations of others' transitions, we also separately examined the other two dimensions of the 3d Mind Model[3,4]: social impact and rationality. For the dimension of social impact, we found a nonsignificant overall linear interaction effect between loneliness and social impact distance, $\beta = -0.016$, $SE = 0.015$, $p = .268$, 95% CI [-0.045, 0.013] (Supplementary Fig. 28A), as well as non-significant quadratic interaction between loneliness and social impact distance, $\beta = 0.027$, $SE = 0.021$, $p = .215$, 95% CI [-0.015, 0.066] (Supplementary Fig. 28B). Similarly, for the dimension of rationality, there was a nonsignificant overall linear interaction effect between loneliness and rationality distance, $\beta = 0.028$, $SE = 0.019$, $p = .155$, 95% CI [-0.010, 0.065] (Supplementary Fig. 29A), and a non-significant quadratic interaction between loneliness and rationality distance, $\beta = 0.012$, $SE = 0.021$, $p = .165$, 95% CI [-0.004, 0.028] (Supplementary Fig. 29B). Given that these interactions were not significant, we did not run post-hoc analyses of marginal estimates.

These findings suggest that our results indicating that lonely individuals expect themselves to be more volatile in the overall 3d Mind Model was primarily driven by the valence dimension, particularly by their tendency to be drawn toward more negative states.

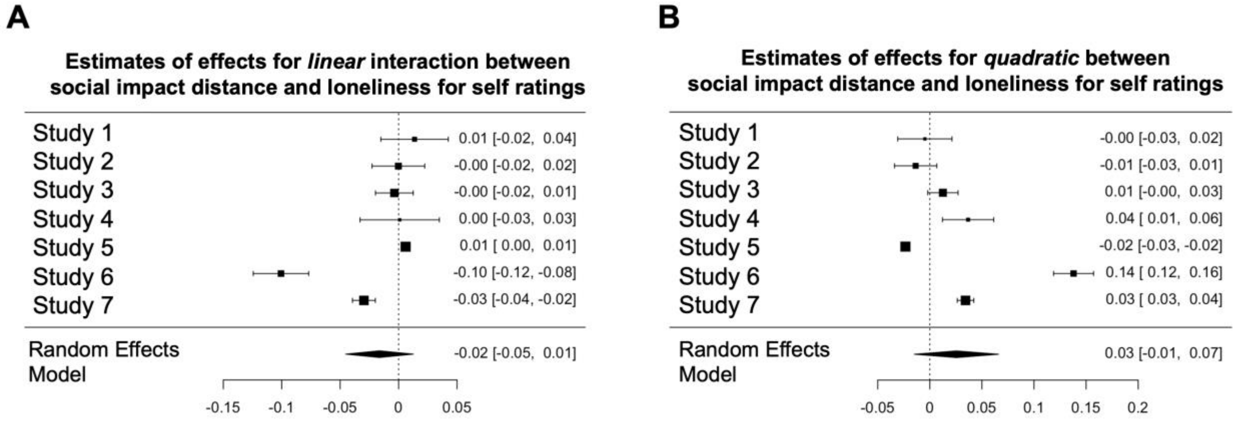

Supplementary Fig. 28

Estimates for Interaction between Social Impact Distance and Loneliness for Self Ratings

(A) Study estimates of the *linear* interaction effect of social impact distance and loneliness for ratings of self emotion transitions. **(B)** Study estimates of the *linear* interaction effect of social impact distance and loneliness for ratings of self emotion transitions. The rhombus at the bottom represents the overall meta-analytic estimate, which is non-significant. Each square represents the estimated interaction effect (standardized) from a single dataset, with error bars indicating the corresponding 95% confidence intervals.

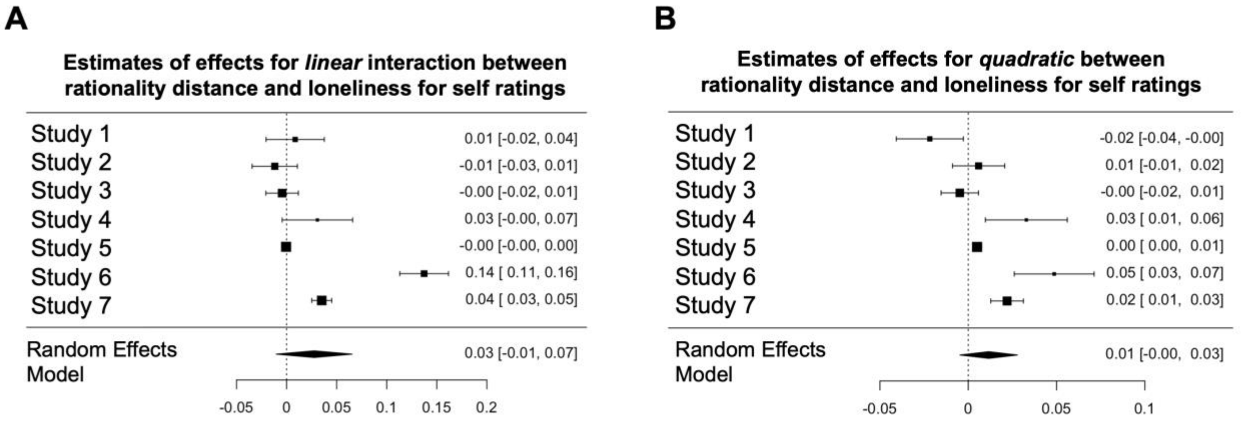

Supplementary Fig. 29

Estimates for Interaction between Rationality Distance and Loneliness for Self Ratings

(A) Study estimates of the *linear* interaction effect of rationality distance and loneliness for ratings of self emotion transitions; **(B)** Study estimates of the *linear* interaction effect of rationality distance and loneliness for ratings of self emotion transitions. The rhombus at the bottom represents the overall meta-analytic estimate, which is non-significant. Each square represents the estimated interaction effect (standardized) from a single dataset, with error bars indicating the corresponding 95% confidence intervals.

Meta Analysis: Confidence for Self Ratings

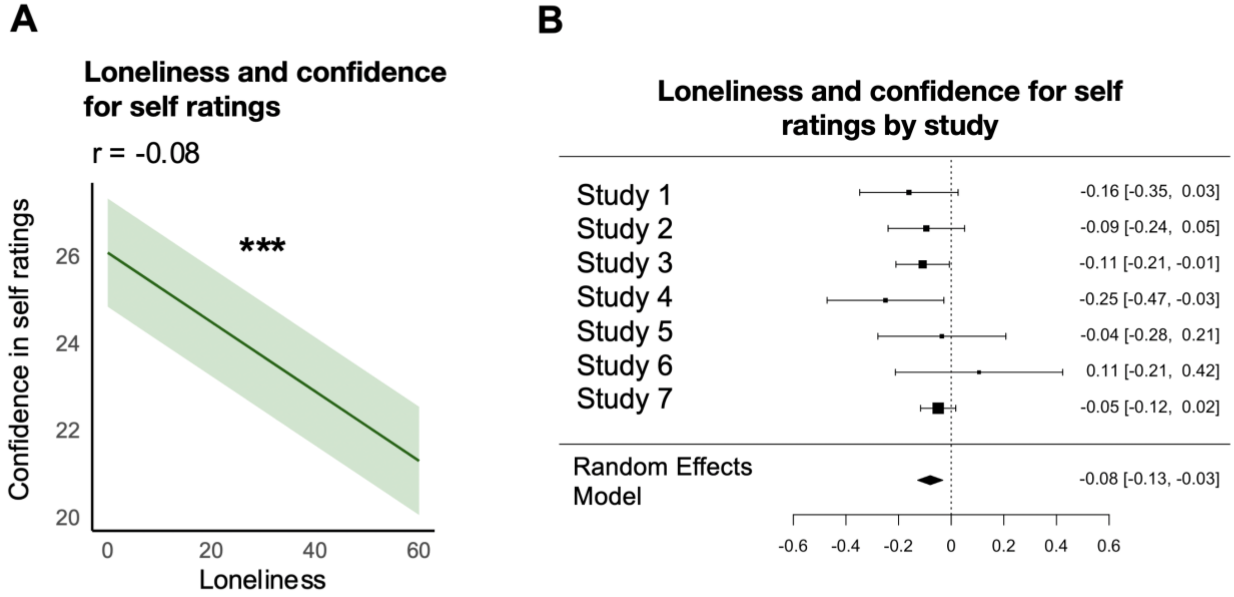

Supplementary Fig. 30

Loneliness is associated with decreased confidence in ratings of one's own emotion transitions

(A) The dark green line represents the meta-analytic correlation estimate and the light green bands indicate the 95% confidence interval. Higher loneliness was associated with lower by-participant standard deviations, suggesting that lonely participants provided less variable ratings. Asterisks indicate significance level: $p < .001$ (***). **(B)** Correlation estimates from individual datasets for the relationship between loneliness and confidence for self ratings. Each square represents the correlation estimate from a single dataset with error bars denoting the corresponding 95% confidence intervals. The rhombus at the bottom represents the overall meta-analytic correlation estimate with its midpoint indicating the average effect size and its width representing the 95% confidence interval, summarizing the effect size across all included studies.

Loneliness and Confidence for Self Ratings by Study

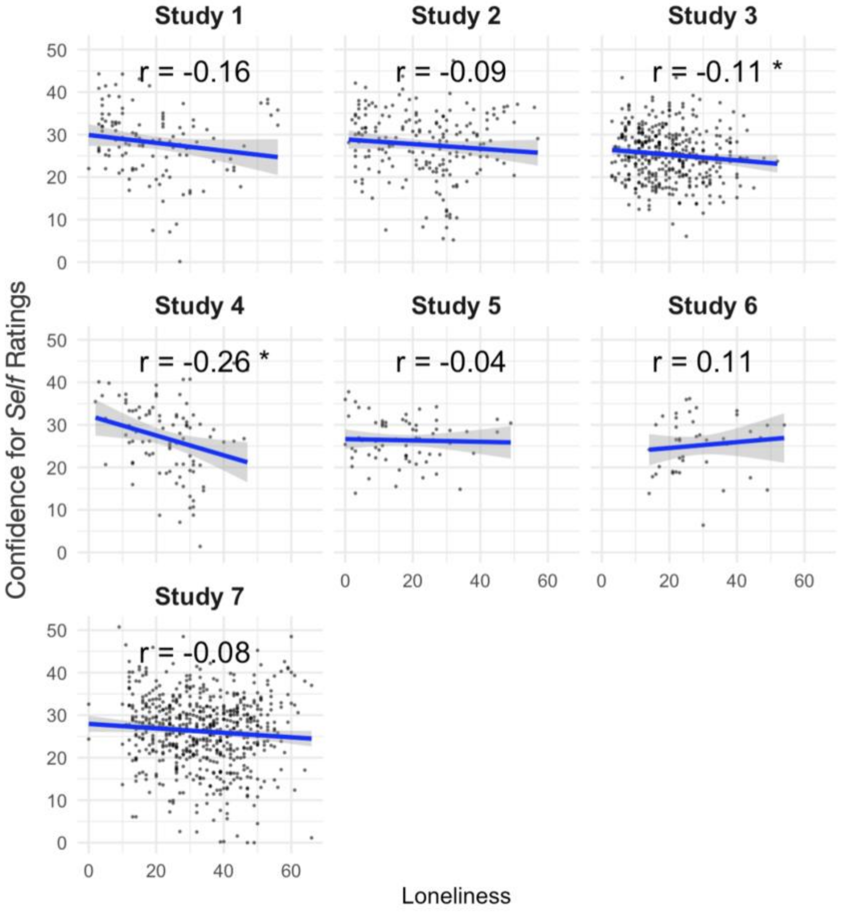

Supplementary Fig. 31

Loneliness and confidence for self ratings by study.

Pearson correlations for the relationship between SD by participant and loneliness for self ratings. *p* values are indicated as follows: *** $p < .001$, **$p < .01$, *$p < .05$.

References

1. Baek, E. C. *et al.* Lonely Individuals Process the World in Idiosyncratic Ways. *Psychol. Sci.* **34**, 683–695 (2023).
2. Finn, E. S. *et al.* Idiosynchrony: From shared responses to individual differences during naturalistic neuroimaging. *NeuroImage* **215**, 116828 (2020).
3. Tamir, D. I., Thornton, M. A., Contreras, J. M. & Mitchell, J. P. Neural evidence that three dimensions organize mental state representation: Rationality, social impact, and valence. *Proc. Natl. Acad. Sci.* **113**, 194–199 (2016).
4. Thornton, M. A. & Tamir, D. I. People represent mental states in terms of rationality, social impact, and valence: Validating the 3d Mind Model. *Cortex* **125**, 44–59 (2020).
